# Employers’ Perspectives on Challenges and Strategies Related To Employment of People with Physical Disabilities: Findings from a National Survey in the United States

**DOI:** 10.1007/s10926-025-10345-9

**Published:** 2025-11-27

**Authors:** Bishan Yang, Emily J. Dinelli, Heerak Choi, Deborah S. Crown, Angelika Kudla, Jasin Wong, Robert Trierweiler, Pamela Capraro, Allen W. Heinemann

**Affiliations:** 1 Shirley Ryan AbilityLab Center for Rehabilitation Outcomes Research, 355 E Erie 11th Floor, Chicago, IL 60611 USA; 2 Department of Special Education, National Tsing Hua University, Hsinchu, Taiwan; 3 Center for Education in Health Sciences, Northwestern University Feinberg School of Medicine, 633 N St. Clair Street 20th Floor, Chicago, IL 60611 USA; 4 Center for Health Services & Outcomes Research, Northwestern University Feinberg School of Medicine, 633 N St. Clair Street 20th Floor, Chicago, IL 60611 USA

**Keywords:** Disability, Employment, Workplace inclusion, Organizational policy

## Abstract

**Purpose:**

The objective of the study was to advance the understanding of employers’ perspectives on challenges and strategies related to hiring, retaining, and promoting people with physical disabilities (PwPD).

**Methods:**

A nationwide cross-sectional survey of 2000 employers was conducted in the US. Descriptive analysis compared disability-related support practices between companies that hired PwPD in the past year and those that did not. Multivariable logistic regression identified specific challenges and strategies associated with inclusive hiring. Additionally, open-text responses were explored using content analysis.

**Results:**

Company characteristics and disability-related support practices differed significantly between companies that hired PwPD (n = 1,142) and those that did not (n = 858). Employers who reported challenges with negative attitudes of coworkers (CI: 0.73–0.94), negative attitudes of customers (CI: 0.73–0.96), not knowing accommodation costs (CI: 0.75–0.98) had decreased odds of hiring PwPD. Conversely, employers who reported implementing onsite technical assistance (CI: 1.18–1.85), ergonomics experts (CI: 1.02–1.59), visible leadership commitment (CI: 1.11–1.73), trial work periods (CI: 1.05–1.63), and universal design (CI: 1.41–2.39) had increased odds. Open-text responses highlighted challenges such as negative attitudes, limited knowledge about how to support PwPD, and concerns about PwPD’s incompetence and litigation. Effective strategies included employee resource groups, skill matching, tailored accommodations, and employee appreciation. Respondents also emphasized the importance of discussing and enhancing disability inclusion.

**Conclusion:**

The study identified specific challenges employers face and strategies they can use when seeking to support the employment of PwPD. Understanding employers’ perspectives reveals opportunities to implement practical strategies that support organizations to enhance the workplace inclusion of PwPD.

**Supplementary Information:**

The online version contains supplementary material available at https://doi.org/10.1007/s10926-025-10345-9.

## Introduction

Despite fluctuations in policy priorities, disability inclusion remains a growing aspect of corporate social responsibility—defined as the broader commitment companies make to bring positive influences on society, communities, and employees [1]—internationally and in the United States (US) [2]. There is increasing recognition of the value of including people with disabilities in the workforce from both institutional and individual perspectives. Corporations that engage in disability-inclusive practices can experience tangible benefits, including improved profitability, employee retention, customer satisfaction, and public reputation [3]. For individuals with disabilities, particularly those with physical disabilities (e.g., stroke, spinal cord injury, spina bifida, multiple sclerosis, limb loss), work participation brings not only financial independence, but also increased socialization, self-worth, mental health, and a stronger sense of societal belongingness [4]. The COVID-19 pandemic and the growth of disability justice movements have amplified awareness of and advocacy for workplace diversity and sparked work opportunities for people living with disabilities. People with physical disabilities (PwPD), in particular, benefit from the expansion of telework that reduces transportation and accessibility barriers [5]; however, significant disparities remain. As of July 2025, the employment–population ratio for working-aged people with disabilities in the US (24.7%) is only about one-third of their non-disabled peers (68.4%) [6]. This gap highlights the unaddressed challenges encountered by people with disabilities throughout their employment journey, from entering the workforce to maintaining jobs to advancing in their careers.

A large body of literature has documented barriers and facilitators to job acquisition and retention reported by PwPD [7–10]. Impairments, such as chronic pain, fatigue, mobility impairments, and comorbidities, can limit the ability to work and maintain job performance. Meanwhile, external supports, such as inclusive workplace policies, accessible environments, job accommodation (e.g., assistive technologies, flexible work schedule), advancement opportunities, and respectful attitudes of coworkers, supervisors, and customers, are critical to job retention and satisfaction. In the absence of these supports, employment of PwPD becomes more challenging. Interventions to promote employment must consider employers’ perspectives, specifically, what motivates or limits organizations from implementing disability-inclusive practices [11].

The importance of exploring employer perspectives on disability inclusion is supported by conceptual frameworks from business studies that highlight key factors influencing human resource management (HRM) practices. Institutional Theory, developed and refined by Scott [12], offers valuable perspectives for understanding how organizational behaviors and practices are shaped by three foundational pillars: the regulative, normative, and cultural-cognitive. The *regulative pillar* encompasses formal rules, laws, and mandates. In the US, regulatory drivers include Sect. 503 of the Rehabilitation Act, which requires federal contractors and subcontractors to ensure equal employment opportunities for individuals with disabilities, and the Americans with Disability Act (ADA) that extends these protections to prohibit discrimination and mandates reasonable accommodations across public and private sectors that have at least 15 employees [13]. The *normative pillar* refers to societal and professional norms that define what is appropriate and ethical in HRM practices, including expectations around diversity and inclusion. The *cultural-cognitive pillar* focuses on the shared beliefs, values, and assumptions, especially those of leadership and HRM practitioners, which influence how disability inclusion is perceived and implemented within a company. Aligned with the cultural-cognitive pillar, inclusive HRM frameworks [14] emphasize the critical role of HRM practitioners in translating regulations and social expectations into actions that support employee inclusion, belongingness, and authenticity. Understanding what enables or hinders leadership and HRM practitioners in supporting disability inclusion is essential for identifying effective strategies at the organizational and structural level.

Although fewer studies focus on employers’ perspectives compared to those centering on the experiences of employees with disabilities, there is a growing body of empirical work in this area. Consistent with the tenets of Institutional Theory, these studies reveal that the decision to hire, retain, and promote people with disabilities is influenced by a mix of organizational structures, individual attitudes, and societal norms. For example, Hulsegge et al. [15] conducted a national survey with over 11,000 organizations in the Netherlands and found that organizations that have employees with disabilities were more likely to adopt inclusive HRM practices. A qualitative study in France [16] revealed that barriers to disability inclusion included negative stereotypes about low productivity and safety risks, concerns about accommodation costs, and administrative burdens in implementing inclusive policies. They also pointed to facilitators, including employers’ motivation, job coach support, and a favorable public sector environment. Comprehensive reviews [17–19] identify a broader set of factors, including organizational-level drivers like leadership commitment, inclusive HR practices, company size, and the belief that disability inclusion contributes to business success.

Despite this evidence, there is still a notable gap in large-scale, US-based research focused on employer perceptions regarding the inclusion of PwPD. The Kessler Foundation’s 2017 and 2022 surveys assessed supervisors’ perspectives on employing people with disabilities [20]. While revealing similar findings as the international literature, they called for increased research efforts on identifying best practices and interventions supporting organizations and leadership in promoting workplace inclusion.

The objective of this study was to advance our understanding of employers’ perspectives on challenges and strategies related to hiring, retaining, and promoting PwPD through a large US national survey. The survey included both closed-ended questions and open-ended questions. Free-text comments are not rare in survey design; however, they are underutilized in formal survey analyses. Open-ended questions offer respondents opportunities to expand on a topic or explain their responses, which can reveal context that closed-ended questions do not [21]. Thus, this study analyzed quantitative and qualitative data to gain a comprehensive appreciation of employers’ perspectives. The specific aims were to: (1) compare employer characteristics, challenges, and strategies between those who have and have not hired PwPD, (2) identify challenges and strategies associated with inclusive hiring, and (3) explore these issues with qualitative responses. This study seeks to fill a gap in the literature by providing large-scale, US-based quantitative and qualitative evidence on employer perceptions of workplace inclusion for PwPD, and offers insights into organizational behaviors, perceived challenges, and strategies, helping to inform approaches to advancing disability inclusion.

## Methods and Materials

### Study Design

We developed a survey to acquire employer-reported company characteristics and HRM practices related to hiring, retaining, and promoting PwPD. Survey design and development drew on job accommodation and disability employment resources [e.g., [21–24], and details have been reported elsewhere [25]. The University Institutional Review Board approved the study (STU00208453).

### Participants and Procedures

We published detailed recruitment procedures for this survey study previously [26]. In brief, we partnered with a market research company to recruit a nationally representative sample of US employers across regions (West: 24%, Midwest: 21%, South: 38%, Northeast: 17%), industries (goods producing: 20%, service providing: 80%), and company sizes (fewer than 500 employees: 50%, 500 or more employees: 50%). Eligible participants were age 18 or older, English-speaking, and involved in HRM responsibilities (e.g., hiring, promoting, providing accommodations, or employee management). Recruitment occurred between April and November 2022, and participants received $25 after completing the survey. The analytic sample of this study included 2000 respondents.

### Measures

#### Quantitative Variables

Hiring history was assessed with the question, “In the past 12 months, has your company hired any people with physical disabilities?” Company factors included years worked at the current job, job title, company size, difficulty retaining employees with physical disabilities, industry type, region, positions that manage requests for accommodations, and processes for employees to declare a disability. Job accommodation-related policies included whether the company: (1) has a process for an employee to request an accommodation, (2) informs employees they can request a reasonable accommodation for disability, (3) has a centralized fund for providing job accommodations, (4) offers alternative accommodations if accommodation requests are denied, (5) trains managers or supervisors on what to do when an employee requests an accommodation, and (6) trains staff regarding job accommodations for retaining and promoting PwPD. Table 1 lists these variables and the response options.

**Table 1.** Sample characteristics

Characteristics	Yes, hired PwPD in the past year (n = 1142)	No, did not hire PwPD in the past year (n = 858)	*P* value
**Company factors**			
Years worked at this job, *mean (SD), years*	7.9 (3.8)	8.4 (4.8)	0.009*
Job title, *n (%)*			0.119
CEO/Owner/President/VP	214 (18.7)	134 (15.6)	
Director/Manager/Admin/Supervisor/Other	208 (18.2)	148 (17.3)	
HR-Related Position	720 (63.1)	576 (67.1)	
Company size, *n (%)*			^a^ < 0.001*
1–49	65 (5.7)	200 (23.3)	
50–499	431 (37.7)	301 (35.1)	
500 +	643 (56.3)	354 (41.3)	
Don’t know	3 (0.3)	3 (0.4)	
Has your company encountered any difficulties retaining employees with physical disabilities?, *n (%)*			^a^ < 0.001*
Yes	445 (39.0)	299 (34.9)	
No	692 (60.6)	535 (62.4)	
Don’t know	5 (0.4)	24 (2.8)	
Industry type, *n (%)*			0.423
Goods producing	236 (20.7)	164 (19.1)	
Service providing	906 (79.3)	694 (80.9)	
Specific industry type, *n (%)*			< 0.001*
Natural resources and mining	27 (2.4)	33 (3.9)	
Construction	80 (7.0)	57 (6.6)	
Manufacturing	129 (11.3)	74 (8.6)	
Trade, transportation, and utilities	176 (15.4)	182 (21.2)	
Information	98 (8.6)	54 (6.3)	
Financial activities	139 (12.2)	96 (11.2)	
Professional and business services	174 (15.2)	169 (19.7)	
Education and health services	192 (16.8)	99 (11.5)	
Leisure and hospitality	113 (9.9)	69 (8.0)	
Other services	14 (1.2)	25 (2.9)	
Region, *n (%)*			< 0.001*
Midwest	265 (23.2)	155 (18.1)	
Northeast	198 (17.3)	142 (16.6)	
South	402 (35.2)	358 (41.7)	
West	277 (24.3)	203 (23.7)	
Who handles requests for accommodations at your company?,* n (%)*			^a^ < 0.001*
Accommodation/ADA specialist	243 (21.3)	100 (11.7)	
CEO/Owner/President/VP/Director of HR/ Employee’s Manager/Supervisor	459 (40.2)	412 (48.0)	
Compliance officer/ HR generalist/ Representative/ Legal department	256 (22.4)	132 (15.4)	
Employee health	77 (6.7)	116 (13.5)	
Employee/Labor relations department	107 (9.4)	94 (11.0)	
Don’t know	0 (0.0)	4 (0.5)	
Does your company have processes for employees to declare disability?, *n (%)*			^a^** < **0.001*
Yes	1090 (95.5)	615 (71.7)	
No	49 (4.3)	230 (26.8)	
Don’t know	3 (0.3)	13 (1.5)	
**Job accommodation-related policies**			
Does your company have a process for an employee to request an accommodation?, *n (%)*			^a^ < 0.001*
Yes	938 (82.1)	514 (59.9)	
No	199 (17.4)	336 (39.2)	
Don’t know	5 (0.4)	8 (0.9)	
Does your company inform employees that they can request a reasonable accommodation for disability?, n (%)			^a^ < 0.001*
Yes	1006 (88.1)	519 (60.5)	
No	129 (11.3)	328 (38.2)	
Don’t know	7 (0.6)	11 (1.3)	
Does your company have a centralized fund for providing job accommodations?, *n (%)*			< 0.001*
Yes	777 (68.0)	266 (31.0)	
No	351 (30.7)	562 (65.5)	
Don’t know	14 (1.2)	30 (3.5)	
If a request for an accommodation is denied, does your company offer an alternative accommodation to the employee?, *n (%)*			< 0.001*
Yes	702 (61.5)	285 (33.2)	
No	430 (37.7)	552 (64.3)	
Don’t know	10 (0.9)	21 (2.5)	
Does your company provide training to managers or supervisors on what to do when an employee requests an accommodation?, *n (%)*			^a^ < 0.001*
Yes	945 (82.8)	467 (54.4)	
No	190 (16.6)	382 (44.5)	
Don’t know	7 (0.6)	9 (1.1)	
Does your company train staff regarding job accommodations for retaining and promoting PwPD?, *n (%)*			< 0.001*
Yes	749 (65.6)	489 (57.0)	
No	382 (33.5)	353 (41.1)	
Don’t know	11 (1.0)	16 (1.9)	

*PwPD* people with physical disabilities, *SD* standard deviation, *n:* number of participants, *%* percentage of participants, *CEO* chief executive officer, *VP* vice president, *ADA* Americans with Disabilities Aact, *HR* human resources. Independent T-tests used for continuous variables;^a^Fisher’s exact test; Chi-square test uses for categorical comparisons otherwise;*Indicates a statistically significant finding *P* < 0.05

Perceived challenges related to retaining and promoting PwPD were assessed (see Table 2). For each challenge, respondents rated each as “very challenging,” “moderately challenging,” “slightly challenging,” “not at all challenging,” or “don’t know.” Strategies that companies used to support retaining and promoting PwPD, including onsite consultation, ergonomics experts, visible leadership commitment, disability awareness training, trial work period, internship programs, tax credits, written corporate policy, use of universal design, and presence of employee resource groups (ERGs), were assessed. Of note, universal design refers to architectural features usable by people of all abilities without the need for adaptation, aiming to ensure accessibility for everyone, including PwPD [27]. Table 3 lists specific strategies.

**Table 2.** Challenges companies experience with retaining and promoting people with physical disabilities

Characteristics	Yes, hired PwPD in the past year (n = 1142)	No, did not hire PwPD in the past year (n = 858)	*P* value
How challenging are the following factors to your company in retaining and promoting people with physical disabilities?			
Lack of knowledge about how to address physical disabilities with employees with disabilities, *n (%)*			^a^ < 0.001*
Very	303 (26.5)	287 (33.5)	
Moderately	483 (42.3)	371 (43.2)	
Slightly	240 (21.0)	148 (17.3)	
Not at all	113 (9.9)	47 (5.5)	
Don’t know	3 (0.3)	5 (0.6)	
Negative attitudes of coworkers, *n (%)*			^a^ < 0.001*
Very	333 (29.2)	370 (43.1)	
Moderately	349 (30.6)	259 (30.2)	
Slightly	310 (27.2)	153 (17.8)	
Not at all	146 (12.8)	64 (7.5)	
Don’t know	4 (0.4)	12 (1.4)	
Negative attitudes of supervisors, *n (%)*			^a^ < 0.001*
Very	328 (28.7)	346 (40.3)	
Moderately	357 (31.3)	285 (33.2)	
Slightly	286 (25.0)	152 (17.7)	
Not at all	171 (15.0)	65 (7.6)	
Don’t know	0 (0.0)	10 (1.2)	
Negative attitudes of customers, *n (%)*			^a^ < 0.001*
Very	288 (25.2)	281 (32.8)	
Moderately	416 (36.4)	350 (40.8)	
Slightly	264 (23.1)	166 (19.4)	
Not at all	169 (14.8)	48 (5.6)	
Don’t know	5 (0.4)	13 (1.5)	
Not knowing how much accommodations will cost, *n (%)*			^a^ < 0.001*
Very	293 (25.7)	320 (37.3)	
Moderately	442 (38.7)	307 (35.8)	
Slightly	287 (25.1)	174 (20.3)	
Not at all	114 (10.0)	49 (5.7)	
Don’t know	6 (0.5)	8 (0.9)	
The cost of providing an accommodation can be too high (unreasonable), *n (%)*			^a^ < 0.001*
Very	393 (34.4)	345 (40.2)	
Moderately	372 (32.6)	293 (34.2)	
Slightly	251 (22.0)	156 (18.2)	
Not at all	122 (10.7)	53 (6.2)	
Don’t know	4 (0.4)	11 (1.3)	
Concern about the cost of healthcare coverage, *n (%)*			^a^0.032*
Very	379 (33.2)	337 (39.3)	
Moderately	430 (37.7)	284 (33.1)	
Slightly	251 (22.0)	176 (20.5)	
Not at all	78 (6.8)	54 (6.3)	
Don’t know	4 (0.4)	7 (0.8)	
Fear of disciplining or firing an employee with disabilities for poor performance, *n (%)*			^a^0.002*
Very	301 (26.4)	281 (32.8)	
Moderately	445 (39.0)	312 (36.4)	
Slightly	310 (27.2)	220 (25.6)	
Not at all	84 (7.4)	40 (4.7)	
Don’t know	2 (0.2)	5 (0.6)	
Concern about litigation (e.g., ADA compliance), *n (%)*			^a^ < 0.001
Very	317 (27.8)	330 (38.5)	
Moderately	436 (38.2)	325 (37.9)	
Slightly	294 (25.7)	148 (17.3)	
Not at all	93 (8.1)	47 (5.5)	
Don’t know	2 (0.2)	8 (0.9)	
Concern about safety in the workplace, *n (%)*			^a^ < 0.001*
Very	337 (29.5)	312 (36.4)	
Moderately	426 (37.3)	349 (40.7)	
Slightly	295 (25.8)	153 (17.8)	
Not at all	81 (7.1)	40 (4.7)	
Don’t know	3 (0.3)	4 (0.5)	
Lack of advancement opportunities for employees with physical disabilities, *n (%)*			^a^ < 0.001*
Very	317 (27.8)	293 (34.2)	
Moderately	420 (36.8)	341 (39.7)	
Slightly	280 (24.5)	153 (17.8)	
Not at all	122 (10.7)	59 (6.9)	
Don’t know	3 (0.3)	12 (1.4)	
Fear that the employee cannot perform the essential functions of the job, *n (%)*			^a^0.002*
Very	382 (33.5)	327 (38.1)	
Moderately	391 (34.2)	297 (34.6)	
Slightly	255 (22.3)	181 (21.1)	
Not at all	112 (9.8)	48 (5.6)	
Don’t know	2 (0.2)	5 (0.6)	

*PwPD* people with physical disabilities, *n* number of participants, *%* percentage of participants, *ADA* Americans with Disabilities Act. Independent T-tests used for continuous variables; ^a^Fisher’s exact test; Chi-square test uses for categorical comparisons otherwise; *Indicates a statistically significant finding *P* < 0.05

**Table 3.** Strategies companies use to retain and promote people with physical disabilities

Characteristics	Yes, hired PwPD in the past year (n = 1142)	No, did not hire PwPD in the past year (n = 858)	*P* value
The following list are strategies that some companies use to retain people with physical disabilities			
Onsite consultation or technical assistance (such as IT, VRC, OT, etc.), *n (%)*			< 0.001*
Yes	700 (61.3)	377 (43.9)	
No	424 (37.1)	471 (54.9)	
Don’t know	18 (1.6)	10 (1.2)	
Bringing in an ergonomics expert for a worksite evaluation, *n (%)*			< 0.001*
Yes	632 (55.3)	367 (42.8)	
No	486 (42.6)	468 (54.6)	
Don’t know	24 (2.1)	23 (2.7)	
Visible leadership commitment, *n (%)*			< 0.001*
Yes	642 (56.2)	341 (39.7)	
No	488 (42.7)	505 (58.9)	
Don’t know	12 (1.1)	12 (1.4)	
Disability awareness training, *n (%)*			< 0.001*
Yes	697 (61.0)	407 (47.4)	
No	427 (37.4)	432 (50.4)	
Don’t know	18 (1.6)	19 (2.2)	
Implementing a trial work period, *n (%)*			< 0.001*
Yes	731 (64.0)	453 (52.8)	
No	388 (34.0)	394 (45.9)	
Don’t know	23 (2.0)	11 (1.3)	
Disability targeted internship programs, *n (%)*			< 0.001*
Yes	635 (55.6)	360 (42.0)	
No	485 (42.5)	482 (56.2)	
Don’t know	22 (1.9)	16 (1.9)	
Employer tax credits and incentives, *n (%)*			< 0.001*
Yes	726 (63.6)	410 (47.8)	
No	393 (34.4)	427 (49.8)	
Don’t know	23 (2.0)	21 (2.5)	
Written corporate policy, *n (%)*			< 0.001*
Yes	789 (69.1)	428 (49.9)	
No	335 (29.3)	415 (48.4)	
Don’t know	18 (1.6)	15 (1.8)	
Does your company incorporate universal design?, *n (%)*			^a^ < 0.001*
Yes	983 (86.1)	475 (55.4)	
No	155 (13.6)	369 (43.0)	
Don’t know	4 (0.4)	14 (1.6)	
Does your company have any employee resources or affinity groups for PwPD?, *n (%)*			^a^ < 0.001*
Yes	767 (67.2)	378 (44.1)	
No	367 (32.1)	461 (53.7)	
Don’t know	8 (0.7)	19 (2.2)	

*PwPD* people with physical disabilities, *n:* number of participants, *%* percentage of participants, *IT* information technology, *VRC*: vocational rehabilitation counselor, *OT* occupational therapist. Independent T-tests used for continuous variables;^a^Fisher’s exact test; Chi-square test uses for categorical comparisons otherwise; *Indicates a statistically significant finding *P* < 0.05

#### Qualitative Data

We analyzed responses to four open-text items: (1) additional challenges regarding employing PwPD not listed in the survey, (2) activities and leadership of disability-related ERGs, (3) additional strategies used by companies, and (4) general comments or feedback about the survey or disability employment.

### Data Analysis

#### Quantitative Analysis

We compared employers who reported hiring PwPD in the past year and those who did not. Employers who responded “don’t know” when asked if their company hired PwPD in the past year were combined with “no” respondents (< 1% of the sample). We calculated descriptive statistics (counts, percentages, means, standard deviations) to summarize variables and compare employer groups; we used Chi-square, Fisher’s exact, and t-tests, as appropriate, to examine group differences in company factors, challenges, and strategies. We used multivariable logistic regression to evaluate the associations between the 12 challenges and 10 strategies with hiring PwPD in the past year. We recoded the challenges response options (0 = no or don’t know, 1 = slightly challenging, 2 = moderately challenging, 3 = very challenging) and strategies (0 = no or don’t know, 1 = yes). All other variables—except for company size—combined “don’t know” responses with “no” responses, after conducting sensitivity analyses treating “don’t know” as missing data and a separate category, respectively. We determined covariates a priori and included job title, company size, industry type, and seven job accommodation-related policies (see Table 1). Each of the 12 challenges and 10 strategies was entered individually in *adjusted* logistic regression models that included 10 covariates. Then, all 12 challenges and 10 strategies were entered into separate *highly adjusted* models that included the same covariates. We conducted quantitative analyses in Microsoft Excel and R version 4.4.1 with listwise deletion of missing cases for regression analyses. Model fits were assessed using Cox-Snell and Nagelkerke pseudo R^2^ and Bayesian Information Criterion (BIC) [28, 29]. Odds ratios, 95% confidence intervals, and* P* values were calculated for each model with *P* values < 0.05 considered as statistically significant.

#### Qualitative Analysis

We used an inductive coding approach to conventional content analysis [30] to explore open-ended responses using ATLAS.ti (version 24) software. Due to the exploratory and descriptive nature of this analysis, we followed a rapid analysis process [31]. Three coders with experience in qualitative research led the analysis. One primary coder (B.Y.) led the development of data-driven code groups, code names, and code definitions, which were reviewed and refined with input from two secondary coders (D.S.C. and R.T.) using the ATLAS.ti coding report. Disagreements were resolved during meetings. All authors were involved in theme development and finalization. The count of responses is reported, but does not represent the generalized frequency of occurrence, nor can it be used to draw inferential conclusions. Rather, it reflects the importance of the content discussed because they were voluntary, free-text responses [32].

## Results

### Participant Characteristics

The sample included 2000 employers with HRM responsibilities (Table 1). Significant differences were found between employers who had hired PwPD in the past year (n = 1142) and those who had not (n = 858) across most company factors and job accommodation policies, except for job title and industry type. Of employers who hired a PwPD in the past year, the years worked at their job was slightly shorter than those who reported not hiring a PwPD in the past year (7.9 years, *P* = 0.009); they worked in companies with 500 + employees (56.3%), reported not encountering difficulties retaining PwPD (60.6%), and worked in service-providing industries (79.3%). Regarding job accommodation policies, those who have hired a PwPD in the past year reported that they had a process for employees to request an accommodation (82.1%), informed employees that they can request a reasonable accommodation for disability (88.1%), had a centralized fund for providing job accommodations (68.0%), offered alternative accommodations to employees whose requests were denied (61.5%), provided training to managers or supervisors on responding to an accommodation request (82.8%), and trained staff regarding job accommodations for retaining and promoting PwPD (65.6%).

### Quantitative Results: Perceived Challenges

Table 2 shows the 12 challenges assessed between employers who hired PwPD in the past year versus those who did not. For all categories, those who did not hire PwPD in the past year more frequently reported greater challenges than those who had. Each challenge was analyzed individually using *adjusted* multivariable logistic regression models that included a single challenge and 10 covariates (See Supplement Tables S1-12). Challenges were treated as numeric values (0, 1, 2, 3) for the purposes of regression modeling. Six of the challenges were significantly associated with a decreased odds of hiring PwPD in the past year: negative attitudes of coworkers (OR: 0.79; 0.71–0.88; *P* < 0.001), negative attitudes of supervisors (OR: 0.89; 0.80–0.99; *P* = 0.035), negative attitudes of customers (OR: 0.81; 0.73–0.91; *P* < 0.001), not knowing the cost of accommodations (OR: 0.82; 0.73–0.92; *P* < 0.001), concerns about litigation (OR: 0.87; 0.78–0.98; *P* = 0.021), and lack of advancement opportunities for PwPD (OR: 0.89; 0.80–1.00; *P* = 0.048).

**Fig. 1:**
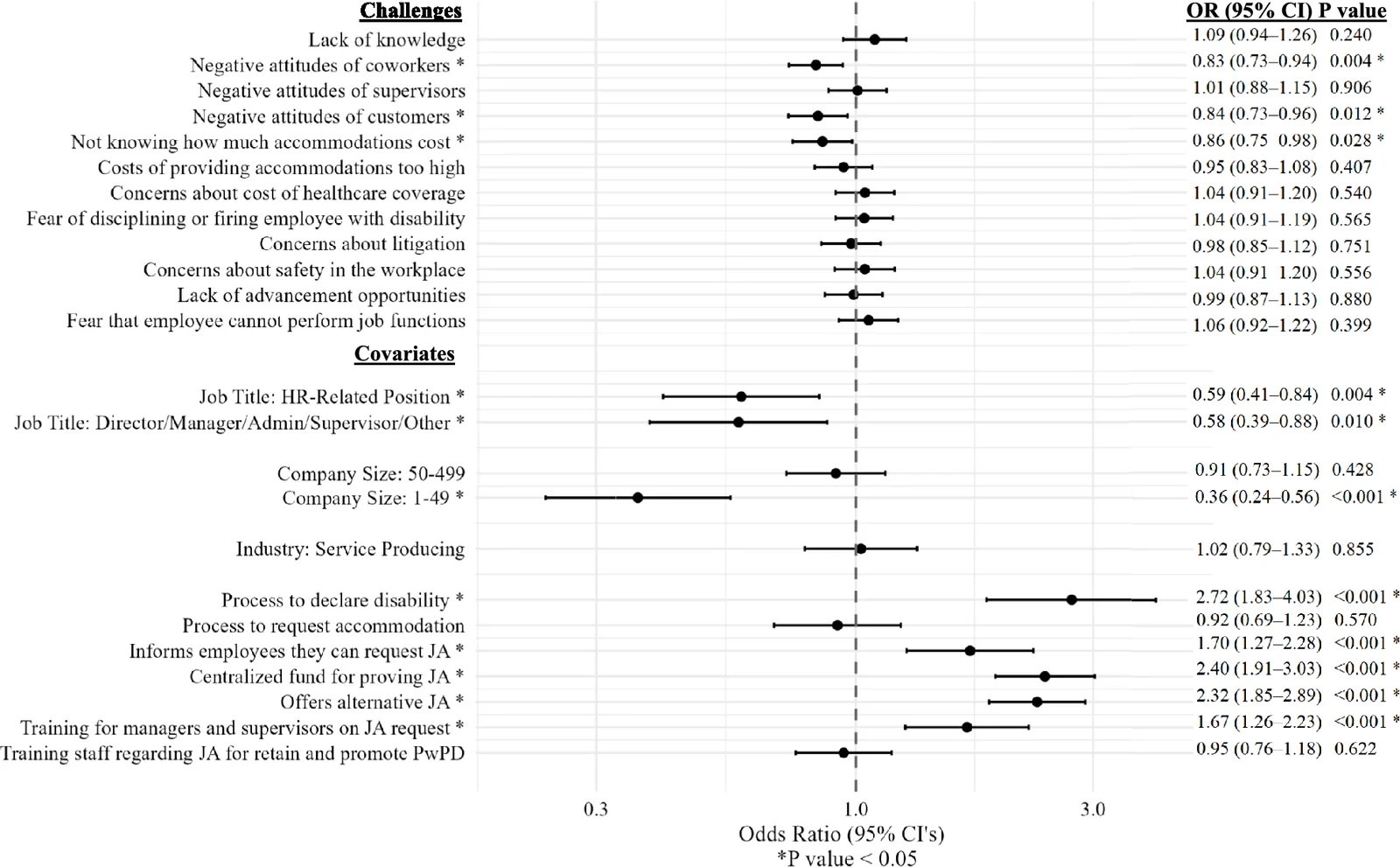
Forest Plot for Highly Adjusted Challenges Model. *JA* job accommodation, *P value < 0.05; PwPD person with physical disability, *OR* odds ratio, *CI* confidence interval, Reference Categories—Challenges: Numeric (0–3); Job title reference group: CEO/Owner/President/VP; Company size reference group: 500 + ; Industry type reference group: Good producing; all other variables reference group: No/Don’t know; Horizontal lines (i.e., 95% CI’s) that cross the vertical dotted line at 1.0 are non-significant. Horizontal lines that do not cross the dotted line at 1.0 are statistically significant at *P* < 0.05. Values to the right of the vertical line represent increased odds of hiring PwPD, while lines to the left of the vertical line represent decreased odds of hiring PwPD

A single, *highly adjusted* multivariable logistic regression model included all challenges and 10 covariates (Fig. 1). Negative attitudes of coworkers (OR: 0.83; 0.73–0.94; *P* = 0.004), negative attitudes of customers (OR: 0.84; 0.73–0.96; *P* = 0.012), and not knowing the cost of accommodations (OR: 0.86; 0.75–0.98; *P* = 0.028) were significantly associated with decreased odds of hiring PwPD. Model fit was appropriate (BIC: 2318.4, Cox-Snell pseudo R^2^: 0.25, Nagelkerke pseudo R^2^: 0.34), and no collinearity was observed with variance inflation factor threshold < 5.

### Quantitative Results: Strategies Used

Table 3 shows the 10 strategies to retain and promote PwPD and compares employers who did vs. did not hire PwPD in the past year. We observed significant group differences for all strategies with a higher proportion of respondents reporting that they used the strategies or supports among those who did vs. did not hire PwPD in the past year. Each strategy and support was individually analyzed using *adjusted* multivariable logistic regression models that included a single strategy or support and the 10 covariates (See Supplement Tables S13-22). All strategies were reported as “yes” if the strategy was used in reference to “no/don’t know.” Onsite consultation or technical assistance (OR: 1.46; 1.18–1.80; *P* < 0.001), visible leadership commitment (OR: 1.36; 1.10–1.68; *P* = 0.005), implementing a trial work period (OR: 1.31; 1.06–1.62; *P* = 0.012), employer tax credits and incentives (OR: 1.25; 1.01–1.55; *P* = 0.041), and incorporating universal design (OR: 1.94; 1.50–2.52; *P* < 0.001) were associated with an increased odds of hiring PwPD.

**Fig. 2:**
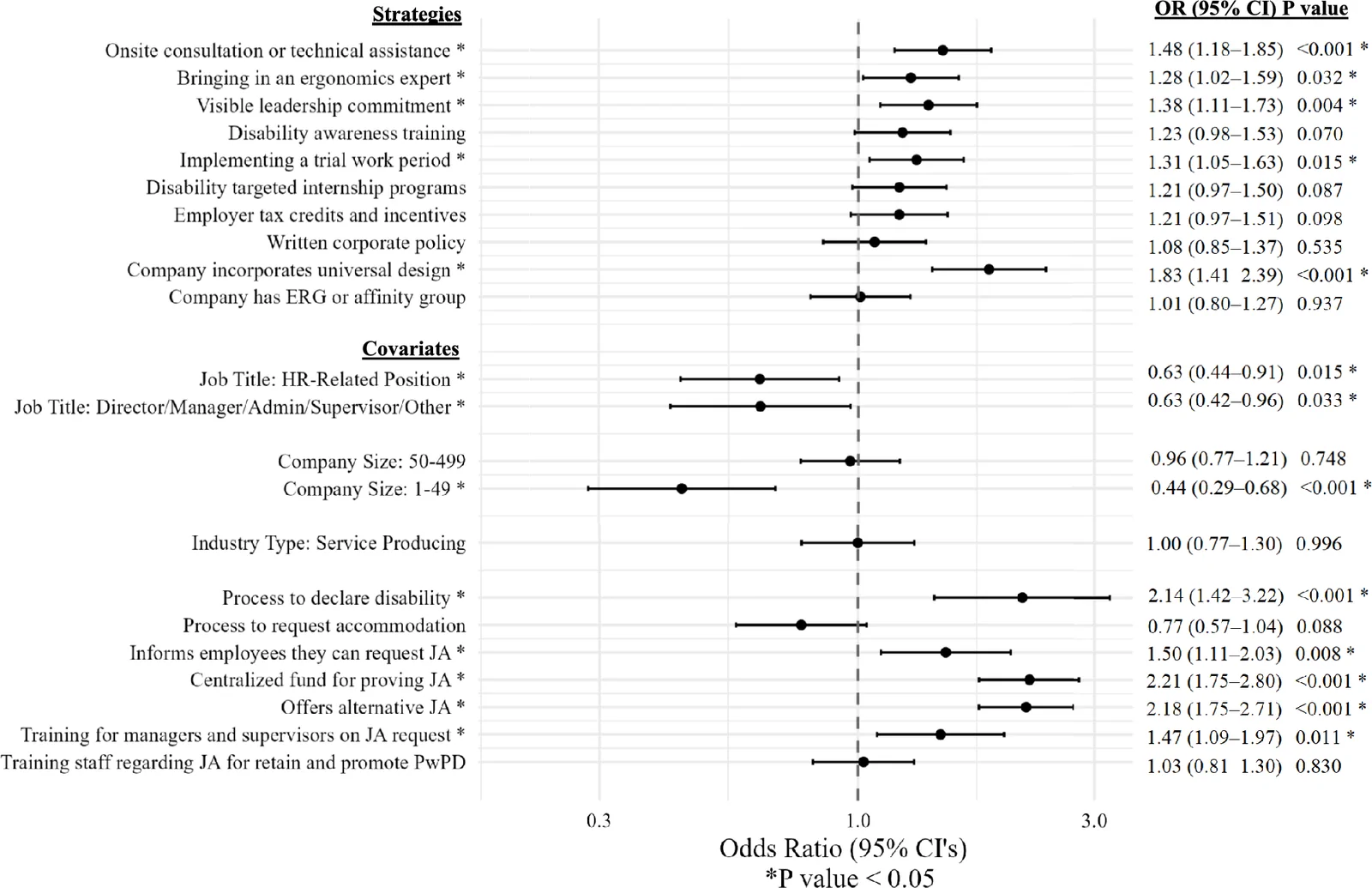
Forest Plot for Highly Adjusted Strategies Model. *JA*: job accommodation, *P value < 0.05; *PwPD* person with physical disability, *OR* odds ratio, *CI* confidence interval, Reference Categories—*Job title reference group*: CEO/Owner/President/VP; *Company size reference group*: 500 + ; *Industry type reference group*: Good producing; *all other variables reference group*: No/Don’t know; Horizontal lines (i.e., 95% CI’s) that cross the vertical dotted line at 1.0 are non-significant. Horizontal lines that do not cross the dotted line at 1.0 are statistically significant at *P* < 0.05. Values to the right of the vertical line represent increased odds of hiring PwPD, while lines to the left of the vertical line represent decreased odds of hiring PwPD

Figure 2 presents results from a single, *highly adjusted* multivariable logistic regression model that included all 10 strategies and the same set of 10 covariates. Onsite consultation or technical assistance (OR: 1.48; 1.18–1.85; *P* < 0.001), bringing in an ergonomics expert (OR: 1.28; 1.02–1.59; *P* = 0.032), visible leadership commitment (OR: 1.38; 1.11–1.73; *P* = 0.004), implementing a trial work period (OR: 1.31; 1.05–1.63; *P* = 0.015), and incorporating universal design (OR: 1.83; 1.41–2.39; *P* < 0.001) were significantly associated with increased odds of hiring PwPD. Overall model fit was appropriate (BIC: 2275.4, Cox-Snell pseudo R^2^: 0.26, Nagelkerke pseudo R^2^: 0.35) and no collinearity was observed with a variance inflation factor threshold of < 5.

### Qualitative Insights

We analyzed 1259 open-text responses to explore employer perspectives on challenges and strategies related to hiring, retaining, and promoting PwPD. Analysis focused on areas of convergence and divergence with the quantitative findings, as well as contextual comments not assessed by the closed-ended questions. Supplement Table S23 presents example responses for each key perspective, which are narrated below.

#### Employers’ Perceived Challenges in Hiring, Retaining, and Promoting PwPD

Of the 256 respondents describing challenges in disability inclusion, some aligned with the quantitative findings about predictors for hiring behavior. First, negative attitudes emerged as a major barrier. Forty-six responses noted stigma and biases from coworkers, customers, and supervisors toward PwPD. These attitudes were perceived as difficult to change and harmful to the workplace climate. Concern about the costs of accommodation was reflected in comments from 15 respondents who expressed fears that job accommodations and healthcare benefits may place financial stress on their organizations.

A broader set of concerns was not shown in the regression results but shared in open-text comments. Concerns about PwPD’s competence were raised in 71 responses, expressing that PwPD may struggle with communication, technology use, and completing tasks on time. Some also had assumptions about cognitive functioning and the ability to meet organizational productivity and performance expectations. Lack of knowledge about how to address PwPD’s needs was expressed in 42 responses. They reflected employers’ insufficient understanding of disability, limited awareness of disability-related needs and support resources. Thirteen respondents expressed fear about potential legal consequences, particularly litigation risks associated with firing employees with disabilities for poor performance.

Finally, open-text responses revealed context-specific challenges not captured by the closed-ended questions. Twenty-three respondents noted that specific challenges in retaining and promoting PwPD may be due to PwPD’s lack of confidence as well as missed opportunities for leadership skill development. Responses also indicated factors influencing the likelihood of overcoming challenges. Sixty-four responses indicated that small and resource-limited companies encounter greater challenges in meeting accessibility needs, navigating regulations, and providing good health benefits than larger companies. Four respondents shared that all of their positions require specific physical functioning abilities that precluded hiring PwPD. One participant noted that supporting the needs of an employee with a disability depends on the person and the specific support they need.

#### Strategies Used to Support Employees with Physical Disabilities

Although having a disability-related ERG was not a predictor in regression analyses, 608 voluntary open-text responses described a range of positive outcomes and the role of ERGs in promoting inclusion. They shared that ERGs enhanced workforce diversity and accessibility, improved job satisfaction, stronger recruitment and retention outcomes, and better service provision for customers with disabilities. Respondents shared that disability-related ERGs serve as a bridge between employees and leadership, facilitating communication about accessibility needs and workplace improvements.

For individual employees, ERGs provide professional development and networking opportunities through meetings, training, mentorship, and events, as reflected by 146 responses. Respondents wrote that ERGs supported employees with disabilities in requesting assistive technologies and job accommodations and created a safe space for employees to voice their concerns. At the organizational and leadership level (96 responses), ERGs were noted to increase disability awareness through education within the company and among external partners. In 69 responses, ERGs were described as advocates for inclusive recruitment and physical, digital, and informational accessibility. ERGs participated in corporate policy-making by reviewing and auditing company practices and identifying opportunities for improvement.

Although ERGs vary in their roles and responsibilities, 48 responses highlighted the importance of leadership involvement in increasing the effectiveness of ERGs, which echoed the significance of visible leadership commitment identified in quantitative data. Respondents indicated that ERGs with executive participation or direct support from senior management had greater visibility and influence, enabling more effective advocacy and policy implementation.

Respondents (216) described four additional strategies. First, 50 responses emphasized routinely assessing and meeting individual accommodation needs, such as modifying tasks, reducing time pressure, and offering adaptive equipment. They highlighted the benefits of providing transportation support. Ten respondents expressed the importance of matching a candidate’s skills with job responsibilities to ensure job fit and performance; 22 described fostering a supportive work environment, emphasizing both physical accessibility and psychological safety. Respect and appreciation (e.g., speaking directly to PwPD, encouraging open communication, and acknowledging their contributions) from colleagues and supervisors were mentioned in 26 responses as key to cultivating belonging.

#### Employers’ Perceived Importance of Workforce Diversity and Inclusion

In the final open-text item, 179 respondents shared reflections. Most respondents praised the survey’s comprehensive design and emphasized the *importance of workplace diversity and inclusion*. Sixteen respondents noted that they learned about new strategies by completing the survey. Additionally, 21 comments described positive experiences with employing PwPD, highlighting the value of inclusion. These respondents emphasized that a diverse workforce is mutually beneficial, not only facilitating business outcomes but also promoting equality, dignity, and meaningful participation in life for individuals with disabilities.

## Discussion

This study compared employer characteristics, challenges, and strategies between those who have and have not hired PwPD in the past year and identified challenges and strategies associated with inclusive hiring and retention. Additional perceived challenges and facilitators from qualitative open-text responses were also explored. Of the challenges assessed, negative attitudes of coworkers, negative attitudes of customers, and not knowing how much job accommodations cost were associated with a decreased likelihood of hiring PwPD in the past year. In contrast, several strategies were associated with an increase in hiring PwPD: utilizing onsite consultation or technical assistance, bringing an ergonomics expert for a worksite evaluation, visible leadership commitment, implementing a trial work period, and incorporating universal design features. Open-text responses were aligned with quantitative findings and revealed additional insights about inclusive employment.

### Challenges Associated with Lower Odds of Hiring PwPD

Aligned with prior literature [10, 15, 25, 33], negative attitudes of coworkers and customers were negatively associated with employers’ hiring of PwPD. Stereotypes and stigma toward disability continue to limit employment opportunities for PwPD. Employers may anticipate negative reactions toward PwPD from coworkers and customers, reinforcing social norms that discourage hiring PwPD and reducing their hiring intention. However, these fears and negative expectations are often not based on evidence, but rather on unfounded assumptions, which create obstacles to hiring and retaining PwPD.

Another barrier to hiring PwPD was employers’ lack of knowledge about the cost of job accommodations. Despite participants’ fears about the costs of accommodations, most accommodations are low or no cost. Some accommodations, such as architectural modifications, qualified interpreters, or advanced technologies, can be expensive; these investments can maximize employees’ functioning and productivity and better align employees’ needs with their work environment [34].

Additional challenges and insights were identified in the open-text responses. Participants expressed concerns about PwPD’s skills, lack of knowledge of how to address PwPD needs, and fears of legal consequences related to employing PwPD. Some participants also explained that the perceived challenges may reflect limited resources and the specific needs of the person with a disability. Small companies typically find it more difficult to meet the needs of employees with disabilities. This speaks to the importance of careful consideration of job assignments based on the skills of PwPD. Successful job placement and matching task assignments are a vital part of ensuring the success of employees with disabilities [35].

### Strategies Associated with Increased Hiring of PwPD

Challenges may persist for employers seeking to hire, retain, and promote PwPD; yet we identified several strategies employers can utilize to promote inclusion. An increase in hiring PwPD was observed among employers that offered onsite consultation or technical assistance and worksite evaluation by an ergonomics expert. Onsite consultations and technical assistance include specific workplace evaluation and planning by professionals such as vocational rehabilitation counselors, occupational therapists, and assistive technology specialists. These professionals can assess the needs of PwPD and develop plans to ensure employees can function in their specific position [9, 36]. Additionally, ergonomics experts can assess a worksite to reduce musculoskeletal injuries. Utilizing professionals with experience in identifying and meeting the needs of PwPD can reduce the burden on employers and enhance employment experiences [37].

A positive association between hiring and the commitment of leadership for retaining and promoting PwPD was observed in the regression model. Open-text respondents highlighted that the positive effects of ERGs were most visible when leadership was involved. Leaders influence both individual attitudes and collective culture [38]. In addition, while having a trial work period is beneficial for all employees and employers by providing a mutual opportunity to assess job fit, it is particularly valued in the context of disability employment [39, 40].

Incorporation of universal design was a significant factor associated with inclusive hiring. Universal design accommodating various needs [27] can add value to workplaces by fostering greater flexibility, enhancing employee satisfaction, and improving productivity for employees regardless of disability. Specific to PwPD, implementing features such as ramps, automatic doors, and ergonomically designed workstations can reduce physical strain and exertion and improve comfort [41]. Companies committed to universal design can enhance their reputation, attract a broader range of customers, improve customer satisfaction, and reduce long-term maintenance costs [42, 43].

### Implications

Our findings offered several practical and policy implications for employers to support workplace inclusion of PwPD. Drawing from the Institutional Theory and inclusive HRM frameworks [12, 14], multi-level efforts are needed within and beyond organizations. As our study findings emphasize corporate efforts to provide reasonable accommodation, policies at the *regulative level* supporting accommodation use can help attract and retain qualified employees, ensure ADA compliance, boost productivity, and foster an inclusive environment [44]. To promote disability-inclusive practices, government agencies can expand tax credits and grants particularly targeting small and resource-limited businesses [45]. The US Department of Labor, JAN, National ADA Network, DisabilityIn, and vocational rehabilitation services can help employers develop sound, compliant practices that minimize fears of litigation and uncertainty around accommodations. Reinforcing ADA compliance through technical assistance and clear accountability mechanisms can also translate legal mandates into action. Employers can take steps to avoid discrimination complaints by defining the essential job functions in written job descriptions, using an interactive process with an employee for selecting an accommodation, and establishing clear policies and procedures for managing job accommodation requests [13]. Finally, public investment and policy support are essential to foster the adoption of costly practices, such as hiring ergonomic experts, providing onsite consultation, and implementing universal design. This is especially important for organizations with limited resources, which our study found face increased challenges in inclusive hiring.

At the normative level, our study indicated that employer training and inter-organizational collaborations play a critical role in fostering workplace inclusion [46]. Findings can inform the development of vocational rehabilitation outreach and professional development programs that emphasize disability awareness, address misconceptions about accommodation costs and productivity, and promote equity-centered HR practices. For example, disability awareness training and executive support for ERGs can address negative attitudes of coworkers and customers, which in turn promote visibility, offer peer support, and advocate for accessible workplace improvements [47, 48]. Visible leadership commitment (e.g., via active engagement in disability-related ERGs and inclusive policy-making processes) may help institutionalize inclusive norms across sectors. In addition, building leader-employee relationships, prioritizing employee well-being, and making inclusive hiring a leadership priority are leadership approaches that can help foster trust, respect, and acceptance of PwPD throughout the organization [46].

Finally, as highlighted in our study on the importance of disability-inclusive climate, shaped by leadership and coworkers, at the *cognitive-cultural level*, HRM practitioners must be equipped with the knowledge and tools to support PwPD [49]. Our findings indicate that stigma and misinformation, particularly regarding accommodation costs and PwPD competence, remain significant barriers. Addressing these requires cultural change through leadership modeling, advocacy from employees, and peer learning. Building internal capacity via consultations with rehabilitation professionals, ergonomic evaluations, and vocational partnerships enables tailored solutions that enhance both individual and organizational performance and organizational readiness. Promoting respect and appreciation, open communication, and psychological safety as workplace culture can reinforce inclusive norms and enable PwPD to contribute meaningfully. These strategies reflect a shift from compliance-based inclusion toward a proactive model of disability-inclusive employment.

### Limitations, Strengths, and Future Directions

Readers should note several limitations. First, the cross-sectional, single-source, and self-report design may introduce same-source bias and limit causal inferences. Employers’ responses may also be subject to social desirability bias as they may feel pressure to present more inclusive perspectives. We asked respondents about their hiring of PwPD in the past year rather than other timeframes. While a one-year timeframe minimizes recall bias and reflects typical hiring behaviors, it limits the generalizability of the findings. Second, several questions had a “don’t know” response option which we combined with “no” responses for most questions with the assumption of the similarity. Although sensitivity analyses treating “don’t know” responses as missing and as a separate category yielded results consistent with the original findings, merging “don’t know” with “no” responses may still obscure nuanced findings. Lastly, we combined industry groups into two categories without exploring specific types.

Despite these limitations, the study has several strengths building on prior similar studies [15–18, 26]. First, many published studies of employer perspectives of workplace inclusion had small samples. We included a large national sample of people with HRM responsibilities holding different job titles. While prior studies focused on the employment of people with general disabilities or intellectual disabilities, this study provides insights relevant to PwPDs. We conducted highly adjusted regression analyses, aiming to identify unique, specific challenges and strategies that inform inclusive, cooperative practices. Lastly, while most survey studies rely solely on quantitative data, we explored open-text responses that provide insights that complement quantitative findings.

Future research should consider longitudinal approaches to identify causality and disaggregate industries for greater granularity. Because open-text responses in our survey were brief and lacked depth, future studies may consider conventional mixed methods designs to collect richer qualitative data. Additionally, investigators should explore intersectional identities and their influence on hiring, retaining, and promoting PwPD. In closing, our large-scale, US-based survey study provides timely, empirical evidence on employers’ perceived challenges and strategies related to inclusive hiring. These findings offer implications across regulative, normative, and cognitive-cultural levels, contributing to the advancement of evidence-informed approaches to workplace inclusion for PwPD.

## Supplementary Information

Below is the link to the electronic supplementary material.

Supplementary file1 (DOCX 86 KB)

Supplementary file2 (DOCX 92 KB)

Supplementary file3 (DOCX 30 KB)

## Data Availability

The data are available from https://www.openicpsr.org/openicpsr/project/213761/version/V1/view
