# Supplementary material for: Employers’ Perspectives on Challenges and Strategies Related To Employment of People with Physical Disabilities: Findings from a National Survey in the United States
Source: J Occup Rehabil. 2025 Nov 27;36(4):1194–209. doi: 10.1007/s10926-025-10345-9 (PMC13759447; doi:10.1007/s10926-025-10345-9)
Supplement: Supplementary file 1 [file 10926_2025_10345_MOESM1_ESM.docx]

**Article Title**: Employers’ perspectives on challenges and strategies related to employment of people with physical disabilities: Findings from a national survey in the United States

**Journal name**: Journal of Occupational Rehabilitation

**Authors**: Bishan Yang, Emily J. Dinelli, Heerak Choi, Deborah S. Crown, Angelika Kudla, Jasin Wong, Robert Trierweiler, Pamela Capraro, Allen W. Heinemann

**Corresponding Authors**:

Bishan Yang, PhD (primary)

Center for Education in Health Sciences

Northwestern University Feinberg School of Medicine

Email: [bishan.yang@northwestern.edu](mailto:heerak.choi@northwestern.edu)

Emily J. Dinelli, MS

Center for Health Services & Outcomes Research

Northwestern University Feinberg School of Medicine

Email: [emily.hurst1@northwestern.edu](mailto:emily.hurst1@northwestern.edu)

Allen W. Heinemann, PhD

Shirley Ryan AbilityLab Center for Rehabilitation Outcomes Research

Email: [aheinemann@sralab.org](mailto:aheinemann@sralab.org)

| **Table S1**. Logistic Regression Results - Lack of Knowledge About How to Address Physical Disabilities with Employees with Disabilities | | | |
| --- | --- | --- | --- |
| Characteristics | OR | 95% CI | *P* value |
| **How challenging are the following factors to your company in retaining and promoting people with physical disabilities?** | | | |
| Lack of knowledge about how to address physical disabilities with employees with disabilities | | | |
| No/Don’t know, Slightly, Moderately, Very (0-3) | 0.98 | 0.87, 1.10 | 0.717 |
| **Covariates** | | | |
| Job title |  |  |  |
| *CEO/Owner/President/VP* | *[Ref]* | *—* |  |
| HR-Related Position | 0.55 | 0.38, 0.78 | <0.001* |
| Director/Manager/Administrator/Supervisor/Other | 0.55 | 0.36, 0.82 | 0.004* |
| Company size |  |  |  |
| *500+* | *[Ref]* | *—* |  |
| 50-499 | 0.94 | 0.75, 1.18 | 0.619 |
| 1-49 | 0.39 | 0.26, 0.59 | <0.001* |
| Industry type |  |  |  |
| *Good Producing* | *[Ref]* | *—* |  |
| Service Producing | 1.03 | 0.80, 1.33 | 0.812 |
| Does your company have processes for employees to declare disability? | | | |
| *No/Don’t know* | *[Ref]* | *—* |  |
| Yes | 2.65 | 1.80, 3.94 | <0.001* |
| Does your company have a process for an employee to request an accommodation? | | | |
| *No/Don’t know* | *[Ref]* | *—* |  |
| Yes | 0.92 | 0.68, 1.22 | 0.552 |
| Does your company inform employees that they can request a reasonable accommodation for disability? | | | |
| *No/Don’t know* | *[Ref]* | *—* |  |
| Yes | 1.82 | 1.36, 2.43 | <0.001* |
| Does your company have a centralized fund for providing job accommodations? | | | |
| *No/Don’t know* | *[Ref]* | *—* |  |
| Yes | 2.44 | 1.94, 3.06 | <0.001* |
| If a request for an accommodation is denied, does your company offer an alternative accommodation to the employee? | | | |
| *No/Don’t know* | *[Ref]* | *—* |  |
| Yes | 2.40 | 1.93, 2.99 | <0.001* |
| Does your company provide training to managers or supervisors on what to do when an employee requests an accommodation? | | | |
| *No/Don’t know* | *[Ref]* | *—* |  |
| Yes | 1.72 | 1.29, 2.28 | <0.001* |
| Does your company train staff regarding job accommodations for retaining and promoting PwPD? | | | |
| *No/Don’t know* | *[Ref]* | *—* |  |
| Yes | 1.01 | 0.81, 1.26 | 0.904 |
| *PwPD*: People with Physical Disabilities; *OR*: Odds Ratio; *CI*: Confidence Interval; *CEO*: Chief Executive Officer; *VP*: Vice President; *HR*: Human Resources; *Ref*: Reference Group  *Indicates a statistically significant finding *P* < 0.05. | | | |

| **Table S2**. Logistic Regression Results – Negative Attitudes of Coworkers | | | |
| --- | --- | --- | --- |
| Characteristics | OR | 95% CI | *P* value |
| **How challenging are the following factors to your company in retaining and promoting people with physical disabilities?** | | | |
| Negative attitudes of coworkers |  |  |  |
| No/Don’t know, Slightly, Moderately, Very (0-3) | 0.79 | 0.71, 0.88 | <0.001* |
| **Covariates** | | | |
| Job Title |  |  |  |
| *CEO/Owner/President/VP* | *[Ref]* | *—* |  |
| HR-Related Position | 0.56 | 0.39, 0.80 | 0.002* |
| Director/Manager/Administrator/Supervisor/Other | 0.55 | 0.37, 0.82 | 0.004* |
| Company Size |  |  |  |
| *500+* | *[Ref]* | *—* |  |
| 50-499 | 0.92 | 0.73, 1.15 | 0.467 |
| 1-49 | 0.38 | 0.25, 0.58 | <0.001* |
| General Industry Type |  |  |  |
| *Good Producing* | *[Ref]* | *—* |  |
| Service Producing | 1.01 | 0.78, 1.31 | 0.931 |
| Process to Declare a Disability |  |  |  |
| *No/Don’t know* | *[Ref]* | *—* |  |
| Yes | 2.72 | 1.84, 4.05 | <0.001* |
| Informing employees they can request job accommodations |  |  |  |
| *No/Don’t know* | *[Ref]* | *—* |  |
| Yes | 1.74 | 1.30, 2.32 | <0.001* |
| Process to request a job accommodation |  |  |  |
| *No/Don’t know* | *[Ref]* | *—* |  |
| Yes | 0.95 | 0.71, 1.27 | 0.725 |
| Centralized fund for job accommodations |  |  |  |
| *No/Don’t know* | *[Ref]* | *—* |  |
| Yes | 2.43 | 1.94, 3.05 | <0.001* |
| Provide alternative job accommodations |  |  |  |
| *No/Don’t know* | *[Ref]* | *—* |  |
| Yes | 2.27 | 1.83, 2.81 | <0.001* |
| Training for managers and supervisors on requesting a job accommodation |  |  |  |
| *No/Don’t know* | *[Ref]* | *—* |  |
| Yes | 1.67 | 1.26, 2.22 | <0.001* |
| Training staff regarding job accommodations for retaining and promoting PwPD |  |  |  |
| *No/Don’t know* | *[Ref]* | *—* |  |
| Yes | 0.96 | 0.77, 1.19 | 0.699 |
| *PwPD*: People with Physical Disabilities; *OR*: Odds Ratio; *CI*: Confidence Interval; *CEO*: Chief Executive Officer; *VP*: Vice President; *HR*: Human Resources; *Ref*: Reference Group  *Indicates a statistically significant finding *P* < 0.05. | | | |

| **Table S3**. Logistic Regression Results – Negative Attitudes of Supervisors | | | |
| --- | --- | --- | --- |
| Characteristics | OR | 95% CI | *P* value |
| **How challenging are the following factors to your company in retaining and promoting people with physical disabilities?** | | | |
| Negative attitudes of supervisors |  |  |  |
| No/Don’t know, Slightly, Moderately, Very (0-3) | 0.89 | 0.80, 0.99 | 0.035* |
| **Covariates** | | | |
| Job Title |  |  |  |
| *CEO/Owner/President/VP* | *[Ref]* | *—* |  |
| HR-Related Position | 0.56 | 0.39, 0.80 | 0.002* |
| Director/Manager/Administrator/Supervisor/Other | 0.54 | 0.36, 0.81 | 0.003* |
| Company Size |  |  |  |
| *500+* | *[Ref]* | *—* |  |
| 50-499 | 0.93 | 0.74, 1.16 | 0.511 |
| 1-49 | 0.38 | 0.25, 0.58 | <0.001* |
| General Industry Type |  |  |  |
| *Good Producing* | *[Ref]* | *—* |  |
| Service Producing | 1.02 | 0.79, 1.32 | 0.862 |
| Process to Declare a Disability |  |  |  |
| *No/Don’t know* | *[Ref]* | *—* |  |
| Yes | 2.64 | 1.79, 3.93 | <0.001* |
| Informing employees they can request job accommodations |  |  |  |
| *No/Don’t know* | *[Ref]* | *—* |  |
| Yes | 1.77 | 1.33, 2.37 | <0.001* |
| Process to request a job accommodation |  |  |  |
| *No/Don’t know* | *[Ref]* | *—* |  |
| Yes | 0.92 | 0.68, 1.23 | 0.563 |
| Centralized fund for job accommodations |  |  |  |
| *No/Don’t know* | *[Ref]* | *—* |  |
| Yes | 2.41 | 1.92, 3.02 | <0.001* |
| Provide alternative job accommodations |  |  |  |
| *No/Don’t know* | *[Ref]* | *—* |  |
| Yes | 2.32 | 1.87, 2.88 | <0.001* |
| Training for managers and supervisors on requesting a job accommodation |  |  |  |
| *No/Don’t know* | *[Ref]* | *—* |  |
| Yes | 1.70 | 1.28, 2.26 | <0.001* |
| Training staff regarding job accommodations for retaining and promoting PwPD |  |  |  |
| *No/Don’t know* | *[Ref]* | *—* |  |
| Yes | 1.00 | 0.80, 1.24 | 0.992 |
| *PwPD*: People with Physical Disabilities; *OR*: Odds Ratio; *CI*: Confidence Interval; *CEO*: Chief Executive Officer; *VP*: Vice President; *HR*: Human Resources; *Ref*: Reference Group  *Indicates a statistically significant finding *P* < 0.05. | | | |

| **Table S4**. Logistic Regression Results – Negative Attitudes of Customers | | | |
| --- | --- | --- | --- |
| Characteristics | OR | 95% CI | *P* value |
| **How challenging are the following factors to your company in retaining and promoting people with physical disabilities?** | | | |
| Negative attitudes of customers |  |  |  |
| No/Don’t know, Slightly, Moderately, Very (0-3) | 0.81 | 0.73, 0.91 | <0.001* |
| **Covariates** | | | |
| Job Title |  |  |  |
| *CEO/Owner/President/VP* | *[Ref]* | *—* |  |
| HR-Related Position | 0.57 | 0.40, 0.81 | 0.002* |
| Director/Manager/Administrator/Supervisor/Other | 0.56 | 0.37, 0.84 | 0.005* |
| Company Size |  |  |  |
| *500+* | *[Ref]* | *—* |  |
| 50-499 | 0.94 | 0.75, 1.18 | 0.580 |
| 1-49 | 0.38 | 0.25, 0.57 | <0.001* |
| General Industry Type |  |  |  |
| *Good Producing* | *[Ref]* | *—* |  |
| Service Producing | 1.01 | 0.78, 1.31 | 0.942 |
| Process to Declare a Disability |  |  |  |
| *No/Don’t know* | *[Ref]* | *—* |  |
| Yes | 2.61 | 1.77, 3.88 | <0.001* |
| Informing employees they can request job accommodations |  |  |  |
| *No/Don’t know* | *[Ref]* | *—* |  |
| Yes | 1.73 | 1.30, 2.31 | <0.001* |
| Process to request a job accommodation |  |  |  |
| *No/Don’t know* | *[Ref]* | *—* |  |
| Yes | 0.90 | 0.67, 1.20 | 0.482 |
| Centralized fund for job accommodations |  |  |  |
| *No/Don’t know* | *[Ref]* | *—* |  |
| Yes | 2.42 | 1.93, 3.04 | <0.001* |
| Provide alternative job accommodations |  |  |  |
| *No/Don’t know* | *[Ref]* | *—* |  |
| Yes | 2.34 | 1.90, 2.90 | <0.001* |
| Training for managers and supervisors on requesting a job accommodation |  |  |  |
| *No/Don’t know* | *[Ref]* | *—* |  |
| Yes | 1.74 | 1.31, 2.31 | <0.001* |
| Training staff regarding job accommodations for retaining and promoting PwPD |  |  |  |
| *No/Don’t know* | *[Ref]* | *—* |  |
| Yes | 0.99 | 0.80, 1.24 | 0.952 |
| *PwPD*: People with Physical Disabilities; *OR*: Odds Ratio; *CI*: Confidence Interval; *CEO*: Chief Executive Officer; *VP*: Vice President; *HR*: Human Resources; *Ref*: Reference Group  *Indicates a statistically significant finding *P* < 0.05. | | | |

| **Table S5**. Logistic Regression Results – Not Knowing How Much Accommodations Will Cost | | | |
| --- | --- | --- | --- |
| Characteristics | OR | 95% CI | *P* value |
| **How challenging are the following factors to your company in retaining and promoting people with physical disabilities?** | | | |
| Not knowing how much accommodations will cost |  |  |  |
| No/Don’t know, Slightly, Moderately, Very (0-3) | 0.82 | 0.73, 0.92 | 0.001* |
| **Covariates** | | | |
| Job Title |  |  |  |
| *CEO/Owner/President/VP* | *[Ref]* | *—* |  |
| HR-Related Position | 0.57 | 0.39, 0.81 | 0.002* |
| Director/Manager/Administrator/Supervisor/Other | 0.58 | 0.38, 0.87 | 0.009* |
| Company Size |  |  |  |
| *500+* | *[Ref]* | *—* |  |
| 50-499 | 0.92 | 0.74, 1.16 | 0.490 |
| 1-49 | 0.37 | 0.24, 0.56 | <0.001* |
| General Industry Type |  |  |  |
| *Good Producing* | *[Ref]* | *—* |  |
| Service Producing | 1.03 | 0.80, 1.33 | 0.805 |
| Process to Declare a Disability |  |  |  |
| *No/Don’t know* | *[Ref]* | *—* |  |
| Yes | 2.74 | 1.86, 4.07 | <0.001* |
| Informing employees they can request job accommodations |  |  |  |
| *No/Don’t know* | *[Ref]* | *—* |  |
| Yes | 1.77 | 1.32, 2.36 | <0.001* |
| Process to request a job accommodation |  |  |  |
| *No/Don’t know* | *[Ref]* | *—* |  |
| Yes | 0.89 | 0.67, 1.19 | 0.441 |
| Centralized fund for job accommodations |  |  |  |
| *No/Don’t know* | *[Ref]* | *—* |  |
| Yes | 2.41 | 1.92, 3.03 | <0.001* |
| Provide alternative job accommodations |  |  |  |
| *No/Don’t know* | *[Ref]* | *—* |  |
| Yes | 2.34 | 1.89, 2.89 | <0.001* |
| Training for managers and supervisors on requesting a job accommodation |  |  |  |
| *No/Don’t know* | *[Ref]* | *—* |  |
| Yes | 1.68 | 1.26, 2.22 | <0.001* |
| Training staff regarding job accommodations for retaining and promoting PwPD |  |  |  |
| *No/Don’t know* | *[Ref]* | *—* |  |
| Yes | 0.99 | 0.79, 1.23 | 0.931 |
| *PwPD*: People with Physical Disabilities; *OR*: Odds Ratio; *CI*: Confidence Interval; *CEO*: Chief Executive Officer; *VP*: Vice President; *HR*: Human Resources; *Ref*: Reference Group  *Indicates a statistically significant finding *P* < 0.05. | | | |

| **Table S6**. Logistic Regression Results - The Cost of Providing an Accommodation Can Be Too High (Unreasonable) | | | |
| --- | --- | --- | --- |
| Characteristics | OR | 95% CI | *P* value |
| **How challenging are the following factors to your company in retaining and promoting people with physical disabilities?** | | | |
| The cost of providing an accommodation can be too high (unreasonable) | | | |
| No/Don’t know, Slightly, Moderately, Very (0-3) | 0.92 | 0.82, 1.02 | 0.130 |
| **Covariates** | | | |
| Job Title |  |  |  |
| *CEO/Owner/President/VP* | *[Ref]* | *—* |  |
| HR-Related Position | 0.55 | 0.38, 0.78 | 0.001* |
| Director/Manager/Administrator/Supervisor/Other | 0.56 | 0.37, 0.84 | 0.005* |
| Company Size |  |  |  |
| *500+* | *[Ref]* | *—* |  |
| 50-499 | 0.94 | 0.75, 1.18 | 0.606 |
| 1-49 | 0.39 | 0.25, 0.59 | <0.001* |
| General Industry Type |  |  |  |
| *Good Producing* | *[Ref]* | *—* |  |
| Service Producing | 1.03 | 0.79, 1.33 | 0.835 |
| Process to Declare a Disability |  |  |  |
| *No/Don’t know* | *[Ref]* | *—* |  |
| Yes | 2.68 | 1.82, 3.98 | <0.001* |
| Informing employees they can request job accommodations |  |  |  |
| *No/Don’t know* | *[Ref]* | *—* |  |
| Yes | 1.79 | 1.34, 2.40 | <0.001* |
| Process to request a job accommodation |  |  |  |
| *No/Don’t know* | *[Ref]* | *—* |  |
| Yes | 0.92 | 0.68, 1.22 | 0.550 |
| Centralized fund for job accommodations |  |  |  |
| *No/Don’t know* | *[Ref]* | *—* |  |
| Yes | 2.45 | 1.96, 3.08 | <0.001* |
| Provide alternative job accommodations |  |  |  |
| *No/Don’t know* | *[Ref]* | *—* |  |
| Yes | 2.37 | 1.92, 2.94 | <0.001* |
| Training for managers and supervisors on requesting a job accommodation |  |  |  |
| *No/Don’t know* | *[Ref]* | *—* |  |
| Yes | 1.69 | 1.27, 2.24 | <0.001* |
| Training staff regarding job accommodations for retaining and promoting PwPD |  |  |  |
| *No/Don’t know* | *[Ref]* | *—* |  |
| Yes | 1.02 | 0.82, 1.26 | 0.883 |
| *PwPD*: People with Physical Disabilities; *OR*: Odds Ratio; *CI*: Confidence Interval; *CEO*: Chief Executive Officer; *VP*: Vice President; *HR*: Human Resources; *Ref*: Reference Group  *Indicates a statistically significant finding *P* < 0.05. | | | |

| **Table S7**. Logistic Regression Results - Concern About the Cost of Health Care Coverage | | | |
| --- | --- | --- | --- |
| Characteristics | OR | 95% CI | *P* value |
| **How challenging are the following factors to your company in retaining and promoting people with physical disabilities?** | | | |
| Concern about the cost of health care coverage |  |  |  |
| No/Don’t know, Slightly, Moderately, Very (0-3) | 0.95 | 0.85, 1.07 | 0.420 |
| **Covariates** | | | |
| Job Title |  |  |  |
| *CEO/Owner/President/VP* | *[Ref]* | *—* |  |
| HR-Related Position | 0.55 | 0.38, 0.78 | <0.001* |
| Director/Manager/Administrator/Supervisor/Other | 0.55 | 0.37, 0.82 | 0.004* |
| Company Size |  |  |  |
| *500+* | *[Ref]* | *—* |  |
| 50-499 | 0.94 | 0.75, 1.18 | 0.584 |
| 1-49 | 0.39 | 0.25, 0.59 | <0.001* |
| General Industry Type |  |  |  |
| *Good Producing* | *[Ref]* | *—* |  |
| Service Producing | 1.03 | 0.79, 1.33 | 0.841 |
| Process to Declare a Disability |  |  |  |
| *No/Don’t know* | *[Ref]* | *—* |  |
| Yes | 2.66 | 1.81, 3.96 | <0.001* |
| Informing employees they can request job accommodations |  |  |  |
| *No/Don’t know* | *[Ref]* | *—* |  |
| Yes | 1.81 | 1.35, 2.41 | <0.001* |
| Process to request a job accommodation |  |  |  |
| *No/Don’t know* | *[Ref]* | *—* |  |
| Yes | 0.91 | 0.68, 1.22 | 0.524 |
| Centralized fund for job accommodations |  |  |  |
| *No/Don’t know* | *[Ref]* | *—* |  |
| Yes | 2.44 | 1.95, 3.07 | <0.001* |
| Provide alternative job accommodations |  |  |  |
| *No/Don’t know* | *[Ref]* | *—* |  |
| Yes | 2.40 | 1.95, 2.97 | <0.001* |
| Training for managers and supervisors on requesting a job accommodation |  |  |  |
| *No/Don’t know* | *[Ref]* | *—* |  |
| Yes | 1.72 | 1.29, 2.28 | <0.001* |
| Training staff regarding job accommodations for retaining and promoting PwPD |  |  |  |
| *No/Don’t know* | *[Ref]* | *—* |  |
| Yes | 1.02 | 0.82, 1.26 | 0.882 |
| *PwPD*: People with Physical Disabilities; *OR*: Odds Ratio; *CI*: Confidence Interval; *CEO*: Chief Executive Officer; *VP*: Vice President; *HR*: Human Resources; *Ref*: Reference Group  *Indicates a statistically significant finding *P* < 0.05. | | | |

| **Table S8**. Logistic Regression Results - Fear of Disciplining or Firing an Employee with Disabilities for Poor Performance | | | |
| --- | --- | --- | --- |
| Characteristics | OR | 95% CI | *P* value |
| **How challenging are the following factors to your company in retaining and promoting people with physical disabilities?** | | | |
| Fear of disciplining or firing an employee with disabilities for poor performance | | | |
| No/Don’t know, Slightly, Moderately, Very (0-3) | 0.97 | 0.86, 1.09 | 0.615 |
| **Covariates** | | | |
| Job Title |  |  |  |
| *CEO/Owner/President/VP* | *[Ref]* | *—* |  |
| HR-Related Position | 0.55 | 0.38, 0.78 | <0.001* |
| Director/Manager/Administrator/Supervisor/Other | 0.55 | 0.36, 0.82 | 0.004* |
| Company Size |  |  |  |
| *500+* | *[Ref]* | *—* |  |
| 50-499 | 0.94 | 0.75, 1.18 | 0.618 |
| 1-49 | 0.39 | 0.26, 0.59 | <0.001* |
| General Industry Type |  |  |  |
| *Good Producing* | *[Ref]* | *—* |  |
| Service Producing | 1.03 | 0.80, 1.33 | 0.808 |
| Process to Declare a Disability |  |  |  |
| *No/Don’t know* | *[Ref]* | *—* |  |
| Yes | 2.65 | 1.80, 3.93 | <0.001* |
| Informing employees they can request job accommodations |  |  |  |
| *No/Don’t know* | *[Ref]* | *—* |  |
| Yes | 1.82 | 1.36, 2.43 | <0.001* |
| Process to request a job accommodation |  |  |  |
| *No/Don’t know* | *[Ref]* | *—* |  |
| Yes | 0.92 | 0.68, 1.22 | 0.558 |
| Centralized fund for job accommodations |  |  |  |
| *No/Don’t know* | *[Ref]* | *—* |  |
| Yes | 2.44 | 1.95, 3.06 | <0.001* |
| Provide alternative job accommodations |  |  |  |
| *No/Don’t know* | *[Ref]* | *—* |  |
| Yes | 2.40 | 1.94, 2.98 | <0.001* |
| Training for managers and supervisors on requesting a job accommodation |  |  |  |
| *No/Don’t know* | *[Ref]* | *—* |  |
| Yes | 1.72 | 1.29, 2.28 | <0.001* |
| Training staff regarding job accommodations for retaining and promoting PwPD |  |  |  |
| *No/Don’t know* | *[Ref]* | *—* |  |
| Yes | 1.01 | 0.81, 1.26 | 0.906 |
| *PwPD*: People with Physical Disabilities; *OR*: Odds Ratio; *CI*: Confidence Interval; *CEO*: Chief Executive Officer; *VP*: Vice President; *HR*: Human Resources; *Ref*: Reference Group  *Indicates a statistically significant finding *P* < 0.05. | | | |

| **Table S9**. Logistic Regression Results - Concern About Litigation (e.g. ADA Compliance) | | | |
| --- | --- | --- | --- |
| Characteristics | OR | 95% CI | *P* value |
| **How challenging are the following factors to your company in retaining and promoting people with physical disabilities?** | | | |
| Concern about litigation (e.g. ADA compliance) |  |  |  |
| No/Don’t know, Slightly, Moderately, Very (0-3) | 0.87 | 0.78, 0.98 | 0.021* |
| **Covariates** | | | |
| Job Title |  |  |  |
| *CEO/Owner/President/VP* | *[Ref]* | *—* |  |
| HR-Related Position | 0.56 | 0.39, 0.80 | 0.002* |
| Director/Manager/Administrator/Supervisor/Other | 0.55 | 0.37, 0.82 | 0.004* |
| Company Size |  |  |  |
| *500+* | *[Ref]* | *—* |  |
| 50-499 | 0.94 | 0.75, 1.18 | 0.610 |
| 1-49 | 0.38 | 0.25, 0.58 | <0.001* |
| General Industry Type |  |  |  |
| *Good Producing* | *[Ref]* | *—* |  |
| Service Producing | 1.02 | 0.79, 1.32 | 0.865 |
| Process to Declare a Disability |  |  |  |
| *No/Don’t know* | *[Ref]* | *—* |  |
| Yes | 2.67 | 1.81, 3.96 | <0.001* |
| Informing employees they can request job accommodations |  |  |  |
| *No/Don’t know* | *[Ref]* | *—* |  |
| Yes | 1.80 | 1.35, 2.41 | <0.001* |
| Process to request a job accommodation |  |  |  |
| *No/Don’t know* | *[Ref]* | *—* |  |
| Yes | 0.91 | 0.68, 1.21 | 0.504 |
| Centralized fund for job accommodations |  |  |  |
| *No/Don’t know* | *[Ref]* | *—* |  |
| Yes | 2.40 | 1.92, 3.02 | <0.001* |
| Provide alternative job accommodations |  |  |  |
| *No/Don’t know* | *[Ref]* | *—* |  |
| Yes | 2.35 | 1.90, 2.90 | <0.001* |
| Training for managers and supervisors on requesting a job accommodation |  |  |  |
| *No/Don’t know* | *[Ref]* | *—* |  |
| Yes | 1.71 | 1.29, 2.27 | <0.001* |
| Training staff regarding job accommodations for retaining and promoting PwPD |  |  |  |
| *No/Don’t know* | *[Ref]* | *—* |  |
| Yes | 1.00 | 0.81, 1.25 | 0.976 |
| *PwPD*: People with Physical Disabilities; *OR*: Odds Ratio; *CI*: Confidence Interval; *CEO*: Chief Executive Officer; *VP*: Vice President; *HR*: Human Resources; *Ref*: Reference Group  *Indicates a statistically significant finding *P* < 0.05. | | | |

| **Table S10**. Logistic Regression Results - Concern About Safety in the Workplace | | | |
| --- | --- | --- | --- |
| Characteristics | OR | 95% CI | *P* value |
| **How challenging are the following factors to your company in retaining and promoting people with physical disabilities?** | | | |
| Concern about safety in the workplace |  |  |  |
| No/Don’t know, Slightly, Moderately, Very (0-3) | 0.89 | 0.79, 1.00 | 0.059 |
| **Covariates** | | | |
| Job Title |  |  |  |
| *CEO/Owner/President/VP* | *[Ref]* | *—* |  |
| HR-Related Position | 0.55 | 0.38, 0.78 | 0.001* |
| Director/Manager/Administrator/Supervisor/Other | 0.55 | 0.37, 0.82 | 0.004* |
| Company Size |  |  |  |
| *500+* | *[Ref]* | *—* |  |
| 50-499 | 0.94 | 0.75, 1.18 | 0.600* |
| 1-49 | 0.39 | 0.26, 0.59 | <0.001* |
| General Industry Type |  |  |  |
| *Good Producing* | *[Ref]* | *—* |  |
| Service Producing | 1.02 | 0.79, 1.32 | 0.890 |
| Process to Declare a Disability |  |  |  |
| *No/Don’t know* | *[Ref]* | *—* |  |
| Yes | 2.72 | 1.84, 4.05 | <0.001* |
| Informing employees they can request job accommodations |  |  |  |
| *No/Don’t know* | *[Ref]* | *—* |  |
| Yes | 1.78 | 1.33, 2.38 | <0.001* |
| Process to request a job accommodation |  |  |  |
| *No/Don’t know* | *[Ref]* | *—* |  |
| Yes | 0.92 | 0.68, 1.22 | 0.560 |
| Centralized fund for job accommodations |  |  |  |
| *No/Don’t know* | *[Ref]* | *—* |  |
| Yes | 2.39 | 1.90, 3.00 | <0.001* |
| Provide alternative job accommodations |  |  |  |
| *No/Don’t know* | *[Ref]* | *—* |  |
| Yes | 2.38 | 1.93, 2.94 | <0.001* |
| Training for managers and supervisors on requesting a job accommodation |  |  |  |
| *No/Don’t know* | *[Ref]* | *—* |  |
| Yes | 1.71 | 1.29, 2.26 | <0.001* |
| Training staff regarding job accommodations for retaining and promoting PwPD |  |  |  |
| *No/Don’t know* | *[Ref]* | *—* |  |
| Yes | 1.01 | 0.81, 1.26 | 0.918 |
| *PwPD*: People with Physical Disabilities; *OR*: Odds Ratio; *CI*: Confidence Interval; *CEO*: Chief Executive Officer; *VP*: Vice President; *HR*: Human Resources; *Ref*: Reference Group  *Indicates a statistically significant finding *P* < 0.05. | | | |

| **Table S11**. Logistic Regression Results - Lack of Advancement Opportunities for Employees with Physical Disabilities | | | |
| --- | --- | --- | --- |
| Characteristics | OR | 95% CI | *P* value |
| **How challenging are the following factors to your company in retaining and promoting people with physical disabilities?** | | | |
| Lack of advancement opportunities for employees with physical disabilities | | | |
| No/Don’t know, Slightly, Moderately, Very (0-3) | 0.89 | 0.80, 1.00 | 0.048* |
| **Covariates** | | | |
| Job Title |  |  |  |
| *CEO/Owner/President/VP* | *[Ref]* | *—* |  |
| HR-Related Position | 0.56 | 0.39, 0.80 | 0.002* |
| Director/Manager/Administrator/Supervisor/Other | 0.57 | 0.38, 0.85 | 0.007* |
| Company Size |  |  |  |
| *500+* | *[Ref]* | *—* |  |
| 50-499 | 0.93 | 0.75, 1.17 | 0.551 |
| 1-49 | 0.38 | 0.25, 0.58 | <0.001* |
| General Industry Type |  |  |  |
| *Good Producing* | *[Ref]* | *—* |  |
| Service Producing | 1.04 | 0.80, 1.34 | 0.786 |
| Process to Declare a Disability |  |  |  |
| *No/Don’t know* | *[Ref]* | *—* |  |
| Yes | 2.65 | 1.80, 3.95 | <0.001* |
| Informing employees they can request job accommodations |  |  |  |
| *No/Don’t know* | *[Ref]* | *—* |  |
| Yes | 1.76 | 1.32, 2.36 | <0.001* |
| Process to request a job accommodation |  |  |  |
| *No/Don’t know* | *[Ref]* | *—* |  |
| Yes | 0.92 | 0.69, 1.23 | 0.574 |
| Centralized fund for job accommodations |  |  |  |
| *No/Don’t know* | *[Ref]* | *—* |  |
| Yes | 2.45 | 1.96, 3.08 | <0.001* |
| Provide alternative job accommodations |  |  |  |
| *No/Don’t know* | *[Ref]* | *—* |  |
| Yes | 2.35 | 1.90, 2.91 | <0.001* |
| Training for managers and supervisors on requesting a job accommodation |  |  |  |
| *No/Don’t know* | *[Ref]* | *—* |  |
| Yes | 1.73 | 1.30, 2.29 | <0.001* |
| Training staff regarding job accommodations for retaining and promoting PwPD |  |  |  |
| *No/Don’t know* | *[Ref]* | *—* |  |
| Yes | 1.01 | 0.81, 1.25 | 0.934 |
| *PwPD*: People with Physical Disabilities; *OR*: Odds Ratio; *CI*: Confidence Interval; *CEO*: Chief Executive Officer; *VP*: Vice President; *HR*: Human Resources; *Ref*: Reference Group  *Indicates a statistically significant finding *P* < 0.05. | | | |

| **Table S12**. Logistic Regression Results - Fear that the Employee Cannot Perform the Essential Functions of the Job | | | |
| --- | --- | --- | --- |
| Characteristics | OR | 95% CI | *P* value |
| **How challenging are the following factors to your company in retaining and promoting people with physical disabilities?** | | | |
| Fear that the employee cannot perform the essential functions of the job | | | |
| No/Don’t know, Slightly, Moderately, Very (0-3) | 0.93 | 0.84, 1.04 | 0.236 |
| **Covariates** | | | |
| Job Title |  |  |  |
| *CEO/Owner/President/VP* | *[Ref]* | *—* |  |
| HR-Related Position | 0.55 | 0.39, 0.79 | 0.001* |
| Director/Manager/Administrator/Supervisor/Other | 0.56 | 0.37, 0.83 | 0.005* |
| Company Size |  |  |  |
| *500+* | *[Ref]* | *—* |  |
| 50-499 | 0.94 | 0.75, 1.18 | 0.584 |
| 1-49 | 0.39 | 0.25, 0.59 | <0.001* |
| General Industry Type |  |  |  |
| *Good Producing* | *[Ref]* | *—* |  |
| Service Producing | 1.02 | 0.79, 1.32 | 0.864 |
| Process to Declare a Disability |  |  |  |
| *No/Don’t know* | *[Ref]* | *—* |  |
| Yes | 2.65 | 1.80, 3.93 | <0.001* |
| Informing employees they can request job accommodations |  |  |  |
| *No/Don’t know* | *[Ref]* | *—* |  |
| Yes | 1.79 | 1.34, 2.40 | <0.001* |
| Process to request a job accommodation |  |  |  |
| *No/Don’t know* | *[Ref]* | *—* |  |
| Yes | 0.91 | 0.68, 1.22 | 0.548 |
| Centralized fund for job accommodations |  |  |  |
| *No/Don’t know* | *[Ref]* | *—* |  |
| Yes | 2.45 | 1.96, 3.08 | <0.001* |
| Provide alternative job accommodations |  |  |  |
| *No/Don’t know* | *[Ref]* | *—* |  |
| Yes | 2.38 | 1.92, 2.94 | <0.001* |
| Training for managers and supervisors on requesting a job accommodation |  |  |  |
| *No/Don’t know* | *[Ref]* | *—* |  |
| Yes | 1.72 | 1.30, 2.28 | <0.001* |
| Training staff regarding job accommodations for retaining and promoting PwPD |  |  |  |
| *No/Don’t know* | *[Ref]* | *—* |  |
| Yes | 1.01 | 0.82, 1.26 | 0.894 |
| *PwPD*: People with Physical Disabilities; *OR*: Odds Ratio; *CI*: Confidence Interval; *CEO*: Chief Executive Officer; *VP*: Vice President; *HR*: Human Resources; *Ref*: Reference Group  *Indicates a statistically significant finding *P* < 0.05. | | | |
