# Supplementary material for: Employers’ Perspectives on Challenges and Strategies Related To Employment of People with Physical Disabilities: Findings from a National Survey in the United States
Source: J Occup Rehabil. 2025 Nov 27;36(4):1194–209. doi: 10.1007/s10926-025-10345-9 (PMC13759447; doi:10.1007/s10926-025-10345-9)
Supplement: Supplementary file 2 [file 10926_2025_10345_MOESM2_ESM.docx]

**Article Title**: Employers’ perspectives on challenges and strategies related to employment of people with physical disabilities: Findings from a national survey in the United States

**Journal name**: Journal of Occupational Rehabilitation

**Authors**: Bishan Yang, Emily J. Dinelli, Heerak Choi, Deborah S. Crown, Angelika Kudla, Jasin Wong, Robert Trierweiler, Pamela Capraro, Allen W. Heinemann

**Corresponding Authors**:

Bishan Yang, PhD (primary)

Center for Education in Health Sciences

Northwestern University Feinberg School of Medicine

Email: [bishan.yang@northwestern.edu](mailto:heerak.choi@northwestern.edu)

Emily J. Dinelli, MS

Center for Health Services & Outcomes Research

Northwestern University Feinberg School of Medicine

Email: [emily.hurst1@northwestern.edu](mailto:emily.hurst1@northwestern.edu)

Allen W. Heinemann, PhD

Shirley Ryan AbilityLab Center for Rehabilitation Outcomes Research

Email: [aheinemann@sralab.org](mailto:aheinemann@sralab.org)

| **Table S13**. Logistic Regression Results - On-Site Consultation or Technical Assistance | | | |
| --- | --- | --- | --- |
| Characteristic | OR | 95% CI | *P* value |
| **The following list are strategies that some companies use to retain people with physical disabilities.** | | | |
| On-site consultation or technical assistance |  |  |  |
| *No/Don’t know* | *[Ref]* | *—* |  |
| Yes | 1.46 | 1.18, 1.80 | <0.001* |
| **Covariates** |  |  |  |
| Job title |  |  |  |
| *CEO/Owner/President/VP* | *[Ref]* | *—* |  |
| HR-Related Position | 0.55 | 0.38, 0.79 | 0.001* |
| Director/Manager/Administrator/Supervisor/Other | 0.55 | 0.37, 0.83 | 0.004* |
| Company size |  |  |  |
| *500+* | *[Ref]* | *—* |  |
| 50-499 | 0.94 | 0.75, 1.18 | 0.581 |
| 1-49 | 0.40 | 0.26, 0.60 | <0.001* |
| Industry type |  |  |  |
| *Good Producing* | *[Ref]* | *—* |  |
| Service Producing | 1.03 | 0.80, 1.33 | 0.813 |
| Does your company have processes for employees to declare disability? | | | |
| *No/Don’t know* | *[Ref]* | *—* |  |
| Yes | 2.59 | 1.75, 3.85 | <0.001* |
| Does your company have a process for an employee to request an accommodation? | | | |
| *No/Don’t know* | *[Ref]* | *—* |  |
| Yes | 0.90 | 0.67, 1.21 | 0.493 |
| Does your company inform employees that they can request a reasonable accommodation for disability? | | | |
| *No/Don’t know* | *[Ref]* | *—* |  |
| Yes | 1.78 | 1.33, 2.38 | <0.001* |
| Does your company have a centralized fund for providing job accommodations? | | | |
| *No/Don’t know* | *[Ref]* | *—* |  |
| Yes | 2.35 | 1.87, 2.95 | <0.001* |
| If a request for an accommodation is denied, does your company offer an alternative accommodation to the employee? | | | |
| *No/Don’t know* | *[Ref]* | *—* |  |
| Yes | 2.41 | 1.95, 2.98 | <0.001* |
| Does your company provide training to managers or supervisors on what to do when an employee requests an accommodation? | | | |
| *No/Don’t know* | *[Ref]* | *—* |  |
| Yes | 1.67 | 1.26, 2.22 | <0.001* |
| Does your company train staff regarding job accommodations for retaining and promoting PwPD? | | | |
| *No/Don’t know* | *[Ref]* | *—* |  |
| Yes | 1.11 | 0.88, 1.38 | 0.376 |
| *PwPD*: People with Physical Disabilities; *OR*: Odds Ratio; *CI*: Confidence Interval; *CEO*: Chief Executive Officer; *VP*: Vice President; *HR*: Human Resources; *Ref*: Reference Group  *Indicates a statistically significant finding *P* < 0.05. | | | |

| **Table S14**. Logistic Regression Results - Bringing in an Ergonomics Expert for a Worksite Evaluation | | | |
| --- | --- | --- | --- |
| Characteristic | OR | 95% CI | *P* value |
| **The following list are strategies that some companies use to retain people with physical disabilities.** | | | |
| Bringing in an ergonomics expert for a worksite evaluation |  |  |  |
| *No/Don’t know* | *[Ref]* | *—* |  |
| Yes | 1.12 | 0.91, 1.38 | 0.279 |
| **Covariates** |  |  |  |
| Job title |  |  |  |
| *CEO/Owner/President/VP* | *[Ref]* | *—* |  |
| HR-Related Position | 0.55 | 0.38, 0.78 | <0.001* |
| Director/Manager/Administrator/Supervisor/Other | 0.55 | 0.36, 0.82 | 0.004* |
| Company size |  |  |  |
| *500+* | *[Ref]* | *—* |  |
| 50-499 | 0.95 | 0.76, 1.18 | 0.626 |
| 1-49 | 0.40 | 0.26, 0.60 | <0.001* |
| Industry type |  |  |  |
| *Good Producing* | *[Ref]* | *—* |  |
| Service Producing | 1.03 | 0.80, 1.33 | 0.815 |
| Does your company have processes for employees to declare disability? | | | |
| *No/Don’t know* | *[Ref]* | *—* |  |
| Yes | 2.62 | 1.78, 3.90 | <0.001* |
| Does your company have a process for an employee to request an accommodation? | | | |
| *No/Don’t know* | *[Ref]* | *—* |  |
| Yes | 0.92 | 0.68, 1.23 | 0.561 |
| Does your company inform employees that they can request a reasonable accommodation for disability? | | | |
| *No/Don’t know* | *[Ref]* | *—* |  |
| Yes | 1.80 | 1.35, 2.41 | <0.001* |
| Does your company have a centralized fund for providing job accommodations? | | | |
| *No/Don’t know* | *[Ref]* | *—* |  |
| Yes | 2.44 | 1.94, 3.06 | <0.001* |
| If a request for an accommodation is denied, does your company offer an alternative accommodation to the employee? | | | |
| *No/Don’t know* | *[Ref]* | *—* |  |
| Yes | 2.41 | 1.95, 2.98 | <0.001* |
| Does your company provide training to managers or supervisors on what to do when an employee requests an accommodation? | | | |
| *No/Don’t know* | *[Ref]* | *—* |  |
| Yes | 1.71 | 1.29, 2.27 | <0.001* |
| Does your company train staff regarding job accommodations for retaining and promoting PwPD? | | | |
| *No/Don’t know* | *[Ref]* | *—* |  |
| Yes | 1.02 | 0.82, 1.26 | 0.885 |
| *PwPD*: People with Physical Disabilities; *OR*: Odds Ratio; *CI*: Confidence Interval; *CEO*: Chief Executive Officer; *VP*: Vice President; *HR*: Human Resources; *Ref*: Reference Group  *Indicates a statistically significant finding *P* < 0.05. | | | |

| **Table S15**. Logistic Regression Results - Visible Leadership Commitment | | | |
| --- | --- | --- | --- |
| Characteristic | OR | 95% CI | *P* value |
| **The following list are strategies that some companies use to retain people with physical disabilities.** | | | |
| Visible leadership commitment |  |  |  |
| *No/Don’t know* | *[Ref]* | *—* |  |
| Yes | 1.36 | 1.10, 1.68 | 0.005* |
| **Covariates** |  |  |  |
| Job title |  |  |  |
| *CEO/Owner/President/VP* | *[Ref]* | *—* |  |
| HR-Related Position | 0.57 | 0.39, 0.80 | 0.002* |
| Director/Manager/Administrator/Supervisor/Other | 0.56 | 0.37, 0.84 | 0.005* |
| Company size |  |  |  |
| *500+* | *[Ref]* | *—* |  |
| 50-499 | 0.95 | 0.76, 1.19 | 0.669 |
| 1-49 | 0.39 | 0.26, 0.59 | <0.001* |
| Industry type |  |  |  |
| *Good Producing* | *[Ref]* | *—* |  |
| Service Producing | 1.02 | 0.79, 1.32 | 0.885 |
| Does your company have processes for employees to declare disability? | | | |
| *No/Don’t know* | *[Ref]* | *—* |  |
| Yes | 2.59 | 1.76, 3.85 | <0.001* |
| Does your company have a process for an employee to request an accommodation? | | | |
| *No/Don’t know* | *[Ref]* | *—* |  |
| Yes | 0.89 | 0.66, 1.19 | 0.423 |
| Does your company inform employees that they can request a reasonable accommodation for disability? | | | |
| *No/Don’t know* | *[Ref]* | *—* |  |
| Yes | 1.82 | 1.36, 2.43 | <0.001* |
| Does your company have a centralized fund for providing job accommodations? | | | |
| *No/Don’t know* | *[Ref]* | *—* |  |
| Yes | 2.48 | 1.98, 3.11 | <0.001* |
| If a request for an accommodation is denied, does your company offer an alternative accommodation to the employee? | | | |
| *No/Don’t know* | *[Ref]* | *—* |  |
| Yes | 2.33 | 1.89, 2.89 | <0.001* |
| Does your company provide training to managers or supervisors on what to do when an employee requests an accommodation? | | | |
| *No/Don’t know* | *[Ref]* | *—* |  |
| Yes | 1.69 | 1.27, 2.24 | <0.001* |
| Does your company train staff regarding job accommodations for retaining and promoting PwPD? | | | |
| *No/Don’t know* | *[Ref]* | *—* |  |
| Yes | 0.98 | 0.78, 1.22 | 0.846 |
| *PwPD*: People with Physical Disabilities; *OR*: Odds Ratio; *CI*: Confidence Interval; *CEO*: Chief Executive Officer; *VP*: Vice President; *HR*: Human Resources; *Ref*: Reference Group  *Indicates a statistically significant finding *P* < 0.05. | | | |

| **Table S16**. Logistic Regression Results - Disability Awareness Training | | | |
| --- | --- | --- | --- |
| Characteristic | OR | 95% CI | *P* value |
| **The following list are strategies that some companies use to retain people with physical disabilities.** | | | |
| Disability awareness training |  |  |  |
| *No/Don’t know* | *[Ref]* | *—* |  |
| Yes | 1.14 | 0.92, 1.40 | 0.240 |
| **Covariates** |  |  |  |
| Job title |  |  |  |
| *CEO/Owner/President/VP* | *[Ref]* | *—* |  |
| HR-Related Position | 0.55 | 0.38, 0.78 | <0.001* |
| Director/Manager/Administrator/Supervisor/Other | 0.55 | 0.36, 0.82 | 0.003 |
| Company size |  |  |  |
| *500+* | *[Ref]* | *—* |  |
| 50-499 | 0.94 | 0.75, 1.18 | 0.582 |
| 1-49 | 0.40 | 0.26, 0.60 | <0.001* |
| Industry type |  |  |  |
| *Good Producing* | *[Ref]* | *—* |  |
| Service Producing | 1.03 | 0.80, 1.33 | 0.803 |
| Does your company have processes for employees to declare disability? | | | |
| *No/Don’t know* | *[Ref]* | *—* |  |
| Yes | 2.61 | 1.77, 3.88 | <0.001* |
| Does your company have a process for an employee to request an accommodation? | | | |
| *No/Don’t know* | *[Ref]* | *—* |  |
| Yes | 0.91 | 0.68, 1.21 | 0.516 |
| Does your company inform employees that they can request a reasonable accommodation for disability? | | | |
| *No/Don’t know* | *[Ref]* | *—* |  |
| Yes | 1.81 | 1.36, 2.42 | <0.001* |
| Does your company have a centralized fund for providing job accommodations? | | | |
| *No/Don’t know* | *[Ref]* | *—* |  |
| Yes | 2.43 | 1.94, 3.05 | <0.001* |
| If a request for an accommodation is denied, does your company offer an alternative accommodation to the employee? | | | |
| *No/Don’t know* | *[Ref]* | *—* |  |
| Yes | 2.41 | 1.95, 2.98 | <0.001* |
| Does your company provide training to managers or supervisors on what to do when an employee requests an accommodation? | | | |
| *No/Don’t know* | *[Ref]* | *—* |  |
| Yes | 1.70 | 1.28, 2.26 | <0.001* |
| Does your company train staff regarding job accommodations for retaining and promoting PwPD? | | | |
| *No/Don’t know* | *[Ref]* | *—* |  |
| Yes | 1.01 | 0.81, 1.26 | 0.924 |
| *PwPD*: People with Physical Disabilities; *OR*: Odds Ratio; *CI*: Confidence Interval; *CEO*: Chief Executive Officer; *VP*: Vice President; *HR*: Human Resources; *Ref*: Reference Group  *Indicates a statistically significant finding *P* < 0.05. | | | |

| **Table S17**. Logistic Regression Results - Implementing a Trial Work Period | | | |
| --- | --- | --- | --- |
| Characteristic | OR | 95% CI | *P* value |
| **The following list are strategies that some companies use to retain people with physical disabilities.** | | | |
| Implementing a trial work period |  |  |  |
| *No/Don’t know* | *[Ref]* | *—* |  |
| Yes | 1.31 | 1.06, 1.62 | 0.012* |
| **Covariates** |  |  |  |
| Job title |  |  |  |
| *CEO/Owner/President/VP* | *[Ref]* | *—* |  |
| HR-Related Position | 0.55 | 0.38, 0.79 | 0.001* |
| Director/Manager/Administrator/Supervisor/Other | 0.55 | 0.36, 0.82 | 0.004* |
| Company size |  |  |  |
| *500+* | *[Ref]* | *—* |  |
| 50-499 | 0.96 | 0.76, 1.20 | 0.699 |
| 1-49 | 0.40 | 0.26, 0.60 | <0.001* |
| Industry type |  |  |  |
| *Good Producing* | *[Ref]* | *—* |  |
| Service Producing | 1.04 | 0.80, 1.34 | 0.772 |
| Does your company have processes for employees to declare disability? | | | |
| *No/Don’t know* | *[Ref]* | *—* |  |
| Yes | 2.60 | 1.77, 3.87 | <0.001* |
| Does your company have a process for an employee to request an accommodation? | | | |
| *No/Don’t know* | *[Ref]* | *—* |  |
| Yes | 0.90 | 0.67, 1.20 | 0.485 |
| Does your company inform employees that they can request a reasonable accommodation for disability? | | | |
| *No/Don’t know* | *[Ref]* | *—* |  |
| Yes | 1.82 | 1.36, 2.43 | <0.001* |
| Does your company have a centralized fund for providing job accommodations? | | | |
| *No/Don’t know* | *[Ref]* | *—* |  |
| Yes | 2.44 | 1.95, 3.06 | <0.001* |
| If a request for an accommodation is denied, does your company offer an alternative accommodation to the employee? | | | |
| *No/Don’t know* | *[Ref]* | *—* |  |
| Yes | 2.40 | 1.94, 2.96 | <0.001* |
| Does your company provide training to managers or supervisors on what to do when an employee requests an accommodation? | | | |
| *No/Don’t know* | *[Ref]* | *—* |  |
| Yes | 1.73 | 1.31, 2.30 | <0.001* |
| Does your company train staff regarding job accommodations for retaining and promoting PwPD? | | | |
| *No/Don’t know* | *[Ref]* | *—* |  |
| Yes | 0.99 | 0.80, 1.24 | 0.957 |
| *PwPD*: People with Physical Disabilities; *OR*: Odds Ratio; *CI*: Confidence Interval; *CEO*: Chief Executive Officer; *VP*: Vice President; *HR*: Human Resources; *Ref*: Reference Group  *Indicates a statistically significant finding *P* < 0.05. | | | |

| **Table S18**. Logistic Regression Results - Disability Targeted Internship Programs | | | |
| --- | --- | --- | --- |
| Characteristic | OR | 95% CI | *P* value |
| **The following list are strategies that some companies use to retain people with physical disabilities.** | | | |
| Disability targeted internship programs |  |  |  |
| *No/Don’t know* | *[Ref]* | *—* |  |
| Yes | 1.20 | 0.97, 1.47 | 0.094 |
| **Covariates** |  |  |  |
| Job title |  |  |  |
| *CEO/Owner/President/VP* | *[Ref]* | *—* |  |
| HR-Related Position | 0.55 | 0.38, 0.78 | 0.001* |
| Director/Manager/Administrator/Supervisor/Other | 0.56 | 0.37, 0.84 | 0.005* |
| Company size |  |  |  |
| *500+* | *[Ref]* | *—* |  |
| 50-499 | 0.94 | 0.75, 1.18 | 0.618 |
| 1-49 | 0.40 | 0.26, 0.60 | <0.001* |
| Industry type |  |  |  |
| *Good Producing* | *[Ref]* | *—* |  |
| Service Producing | 1.03 | 0.79, 1.33 | 0.834 |
| Does your company have processes for employees to declare disability? | | | |
| *No/Don’t know* | *[Ref]* | *—* |  |
| Yes | 2.59 | 1.75, 3.85 | <0.001* |
| Does your company have a process for an employee to request an accommodation? | | | |
| *No/Don’t know* | *[Ref]* | *—* |  |
| Yes | 0.91 | 0.68, 1.22 | 0.531 |
| Does your company inform employees that they can request a reasonable accommodation for disability? | | | |
| *No/Don’t know* | *[Ref]* | *—* |  |
| Yes | 1.80 | 1.34, 2.40 | <0.001* |
| Does your company have a centralized fund for providing job accommodations? | | | |
| *No/Don’t know* | *[Ref]* | *—* |  |
| Yes | 2.42 | 1.93, 3.04 | <0.001* |
| If a request for an accommodation is denied, does your company offer an alternative accommodation to the employee? | | | |
| *No/Don’t know* | *[Ref]* | *—* |  |
| Yes | 2.40 | 1.95, 2.97 | <0.001* |
| Does your company provide training to managers or supervisors on what to do when an employee requests an accommodation? | | | |
| *No/Don’t know* | *[Ref]* | *—* |  |
| Yes | 1.73 | 1.31, 2.30 | <0.001* |
| Does your company train staff regarding job accommodations for retaining and promoting PwPD? | | | |
| *No/Don’t know* | *[Ref]* | *—* |  |
| Yes | 1.01 | 0.81, 1.25 | 0.962 |
| *PwPD*: People with Physical Disabilities; *OR*: Odds Ratio; *CI*: Confidence Interval; *CEO*: Chief Executive Officer; *VP*: Vice President; *HR*: Human Resources; *Ref*: Reference Group  *Indicates a statistically significant finding *P* < 0.05. | | | |

| **Table S19**. Logistic Regression Results - Employer Tax Credits and Incentives | | | |
| --- | --- | --- | --- |
| Characteristic | OR | 95% CI | *P* value |
| **The following list are strategies that some companies use to retain people with physical disabilities.** | | | |
| Employer tax credits and incentives |  |  |  |
| *No/Don’t know* | *[Ref]* | *—* |  |
| Yes | 1.25 | 1.01, 1.55 | 0.041* |
| **Covariates** |  |  |  |
| Job title |  |  |  |
| *CEO/Owner/President/VP* | *[Ref]* | *—* |  |
| HR-Related Position | 0.55 | 0.38, 0.78 | 0.001* |
| Director/Manager/Administrator/Supervisor/Other | 0.55 | 0.36, 0.82 | 0.003* |
| Company size |  |  |  |
| *500+* | *[Ref]* | *—* |  |
| 50-499 | 0.94 | 0.75, 1.17 | 0.560 |
| 1-49 | 0.40 | 0.26, 0.60 | <0.001* |
| Industry type |  |  |  |
| *Good Producing* | *[Ref]* | *—* |  |
| Service Producing | 1.03 | 0.80, 1.34 | 0.794 |
| Does your company have processes for employees to declare disability? | | | |
| *No/Don’t know* | *[Ref]* | *—* |  |
| Yes | 2.58 | 1.75, 3.84 | <0.001* |
| Does your company have a process for an employee to request an accommodation? | | | |
| *No/Don’t know* | *[Ref]* | *—* |  |
| Yes | 0.90 | 0.67, 1.20 | 0.471 |
| Does your company inform employees that they can request a reasonable accommodation for disability? | | | |
| *No/Don’t know* | *[Ref]* | *—* |  |
| Yes | 1.81 | 1.36, 2.42 | <0.001* |
| Does your company have a centralized fund for providing job accommodations? | | | |
| *No/Don’t know* | *[Ref]* | *—* |  |
| Yes | 2.45 | 1.96, 3.08 | <0.001* |
| If a request for an accommodation is denied, does your company offer an alternative accommodation to the employee? | | | |
| *No/Don’t know* | *[Ref]* | *—* |  |
| Yes | 2.40 | 1.95, 2.97 | <0.001* |
| Does your company provide training to managers or supervisors on what to do when an employee requests an accommodation? | | | |
| *No/Don’t know* | *[Ref]* | *—* |  |
| Yes | 1.68 | 1.26, 2.23 | <0.001* |
| Does your company train staff regarding job accommodations for retaining and promoting PwPD? | | | |
| *No/Don’t know* | *[Ref]* | *—* |  |
| Yes | 1.00 | 0.80, 1.24 | 0.977 |
| *PwPD*: People with Physical Disabilities; *OR*: Odds Ratio; *CI*: Confidence Interval; *CEO*: Chief Executive Officer; *VP*: Vice President; *HR*: Human Resources; *Ref*: Reference Group  *Indicates a statistically significant finding *P* < 0.05. | | | |

| **Table S20**. Logistic Regression Results - Written Corporate Policy | | | |
| --- | --- | --- | --- |
| Characteristic | OR | 95% CI | *P* value |
| **The following list are strategies that some companies use to retain people with physical disabilities.** | | | |
| Written corporate policy |  |  |  |
| *No/Don’t know* | *[Ref]* | *—* |  |
| Yes | 1.12 | 0.89, 1.41 | 0.326 |
| **Covariates** |  |  |  |
| Job title |  |  |  |
| *CEO/Owner/President/VP* | *[Ref]* | *—* |  |
| HR-Related Position | 0.55 | 0.38, 0.78 | 0.001* |
| Director/Manager/Administrator/Supervisor/Other | 0.55 | 0.36, 0.82 | 0.003* |
| Company size |  |  |  |
| *500+* | *[Ref]* | *—* |  |
| 50-499 | 0.94 | 0.75, 1.18 | 0.589 |
| 1-49 | 0.39 | 0.26, 0.60 | <0.001* |
| Industry type |  |  |  |
| *Good Producing* | *[Ref]* | *—* |  |
| Service Producing | 1.03 | 0.80, 1.33 | 0.821 |
| Does your company have processes for employees to declare disability? | | | |
| *No/Don’t know* | *[Ref]* | *—* |  |
| Yes | 2.59 | 1.75, 3.86 | <0.001* |
| Does your company have a process for an employee to request an accommodation? | | | |
| *No/Don’t know* | *[Ref]* | *—* |  |
| Yes | 0.90 | 0.67, 1.21 | 0.483 |
| Does your company inform employees that they can request a reasonable accommodation for disability? | | | |
| *No/Don’t know* | *[Ref]* | *—* |  |
| Yes | 1.79 | 1.34, 2.39 | <0.001* |
| Does your company have a centralized fund for providing job accommodations? | | | |
| *No/Don’t know* | *[Ref]* | *—* |  |
| Yes | 2.44 | 1.95, 3.06 | <0.001* |
| If a request for an accommodation is denied, does your company offer an alternative accommodation to the employee? | | | |
| *No/Don’t know* | *[Ref]* | *—* |  |
| Yes | 2.41 | 1.95, 2.98 | <0.001* |
| Does your company provide training to managers or supervisors on what to do when an employee requests an accommodation? | | | |
| *No/Don’t know* | *[Ref]* | *—* |  |
| Yes | 1.70 | 1.28, 2.26 | <0.001* |
| Does your company train staff regarding job accommodations for retaining and promoting PwPD? | | | |
| *No/Don’t know* | *[Ref]* | *—* |  |
| Yes | 1.00 | 0.80, 1.25 | 0.988 |
| *PwPD*: People with Physical Disabilities; *OR*: Odds Ratio; *CI*: Confidence Interval; *CEO*: Chief Executive Officer; *VP*: Vice President; *HR*: Human Resources; *Ref*: Reference Group  *Indicates a statistically significant finding *P* < 0.05. | | | |

| **Table S21**. Logistic Regression Results – Company Incorporates Universal Design | | | |
| --- | --- | --- | --- |
| Characteristic | OR | 95% CI | *P* value |
| **The following list are strategies that some companies use to retain people with physical disabilities.** | | | |
| Company incorporates universal design |  |  |  |
| *No/Don’t know* | *[Ref]* | *—* |  |
| Yes | 1.94 | 1.50, 2.52 | <0.001* |
| **Covariates** |  |  |  |
| Job title |  |  |  |
| *CEO/Owner/President/VP* | *[Ref]* | *—* |  |
| HR-Related Position | 0.60 | 0.42, 0.85 | 0.005* |
| Director/Manager/Administrator/Supervisor/Other | 0.60 | 0.40, 0.91 | 0.016* |
| Company size |  |  |  |
| *500+* | *[Ref]* | *—* |  |
| 50-499 | 0.96 | 0.76, 1.20 | 0.705 |
| 1-49 | 0.42 | 0.27, 0.64 | <0.001* |
| Industry type |  |  |  |
| *Good Producing* | *[Ref]* | *—* |  |
| Service Producing | 1.02 | 0.79, 1.32 | 0.874 |
| Does your company have processes for employees to declare disability? | | | |
| *No/Don’t know* | *[Ref]* | *—* |  |
| Yes | 2.45 | 1.66, 3.66 | <0.001* |
| Does your company have a process for an employee to request an accommodation? | | | |
| *No/Don’t know* | *[Ref]* | *—* |  |
| Yes | 0.86 | 0.64, 1.15 | 0.312 |
| Does your company inform employees that they can request a reasonable accommodation for disability? | | | |
| *No/Don’t know* | *[Ref]* | *—* |  |
| Yes | 1.65 | 1.23, 2.21 | <0.001* |
| Does your company have a centralized fund for providing job accommodations? | | | |
| *No/Don’t know* | *[Ref]* | *—* |  |
| Yes | 2.24 | 1.78, 2.82 | <0.001* |
| If a request for an accommodation is denied, does your company offer an alternative accommodation to the employee? | | | |
| *No/Don’t know* | *[Ref]* | *—* |  |
| Yes | 2.36 | 1.91, 2.92 | <0.001* |
| Does your company provide training to managers or supervisors on what to do when an employee requests an accommodation? | | | |
| *No/Don’t know* | *[Ref]* | *—* |  |
| Yes | 1.60 | 1.20, 2.14 | 0.001* |
| Does your company train staff regarding job accommodations for retaining and promoting PwPD? | | | |
| *No/Don’t know* | *[Ref]* | *—* |  |
| Yes | 1.03 | 0.83, 1.28 | 0.786 |
| *PwPD*: People with Physical Disabilities; *OR*: Odds Ratio; *CI*: Confidence Interval; *CEO*: Chief Executive Officer; *VP*: Vice President; *HR*: Human Resources; *Ref*: Reference Group  *Indicates a statistically significant finding *P* < 0.05. | | | |

| **Table S22**. Logistic Regression Results – Employee Resource of Affinity Group for People with Physical Disabilities | | | |
| --- | --- | --- | --- |
| Characteristic | OR | 95% CI | *P* value |
| **The following list are strategies that some companies use to retain people with physical disabilities.** | | | |
| Company has any employee resources or affinity groups for PwPD | | | |
| *No/Don’t know* | *[Ref]* | *—* |  |
| Yes | 1.01 | 0.80, 1.26 | 0.949 |
| **Covariates** |  |  |  |
| Job title |  |  |  |
| *CEO/Owner/President/VP* | *[Ref]* | *—* |  |
| HR-Related Position | 0.55 | 0.38, 0.78 | <0.001* |
| Director/Manager/Administrator/Supervisor/Other | 0.55 | 0.36, 0.82 | 0.004* |
| Company size |  |  |  |
| *500+* | *[Ref]* | *—* |  |
| 50-499 | 0.94 | 0.75, 1.18 | 0.614 |
| 1-49 | 0.39 | 0.26, 0.60 | <0.001* |
| Industry type |  |  |  |
| *Good Producing* | *[Ref]* | *—* |  |
| Service Producing | 1.03 | 0.80, 1.33 | 0.799 |
| Does your company have processes for employees to declare disability? | | | |
| *No/Don’t know* | *[Ref]* | *—* |  |
| Yes | 2.64 | 1.78, 3.94 | <0.001* |
| Does your company have a process for an employee to request an accommodation? | | | |
| *No/Don’t know* | *[Ref]* | *—* |  |
| Yes | 0.92 | 0.68, 1.23 | 0.558 |
| Does your company inform employees that they can request a reasonable accommodation for disability? | | | |
| *No/Don’t know* | *[Ref]* | *—* |  |
| Yes | 1.82 | 1.36, 2.43 | <0.001* |
| Does your company have a centralized fund for providing job accommodations? | | | |
| *No/Don’t know* | *[Ref]* | *—* |  |
| Yes | 2.44 | 1.94, 3.06 | <0.001* |
| If a request for an accommodation is denied, does your company offer an alternative accommodation to the employee? | | | |
| *No/Don’t know* | *[Ref]* | *—* |  |
| Yes | 2.42 | 1.96, 3.00 | <0.001* |
| Does your company provide training to managers or supervisors on what to do when an employee requests an accommodation? | | | |
| *No/Don’t know* | *[Ref]* | *—* |  |
| Yes | 1.72 | 1.29, 2.29 | <0.001* |
| Does your company train staff regarding job accommodations for retaining and promoting PwPD? | | | |
| *No/Don’t know* | *[Ref]* | *—* |  |
| Yes | 1.02 | 0.82, 1.26 | 0.884 |
| *PwPD*: People with Physical Disabilities; *OR*: Odds Ratio; *CI*: Confidence Interval; *CEO*: Chief Executive Officer; *VP*: Vice President; *HR*: Human Resources; *Ref*: Reference Group  *Indicates a statistically significant finding *P* < 0.05. | | | |
