# Supplementary material for: Employers’ Perspectives on Challenges and Strategies Related To Employment of People with Physical Disabilities: Findings from a National Survey in the United States
Source: J Occup Rehabil. 2025 Nov 27;36(4):1194–209. doi: 10.1007/s10926-025-10345-9 (PMC13759447; doi:10.1007/s10926-025-10345-9)
Supplement: Supplementary file 3 [file 10926_2025_10345_MOESM3_ESM.docx]

**Article Title**: Employers’ perspectives on challenges and strategies related to employment of people with physical disabilities: Findings from a national survey in the United States

**Journal name**: Journal of Occupational Rehabilitation

**Authors**: Bishan Yang, Emily J. Dinelli, Heerak Choi, Deborah S. Crown, Angelika Kudla, Jasin Wong, Robert Trierweiler, Pamela Capraro, Allen W. Heinemann

**Corresponding Authors**:

Bishan Yang, PhD (primary)

Center for Education in Health Sciences

Northwestern University Feinberg School of Medicine

Email: [bishan.yang@northwestern.edu](mailto:heerak.choi@northwestern.edu)

Emily J. Dinelli, MS

Center for Health Services & Outcomes Research

Northwestern University Feinberg School of Medicine

Email: [emily.hurst1@northwestern.edu](mailto:emily.hurst1@northwestern.edu)

Allen W. Heinemann, PhD

Shirley Ryan AbilityLab Center for Rehabilitation Outcomes Research

Email: [aheinemann@sralab.org](mailto:aheinemann@sralab.org)

| **Table S23. Summary of Qualitative Insights** | |
| --- | --- |
| **Construct** | **Example Statements** |
| **Challenges** | |
| Negative attitudes | “To create a comfortable and safe environment for people with disabilities is quite difficult because of negative attitude of some workers and customers towards them.”  “We are a small private business so it can be financially challenging to cover the cost of accommodations but we always do so. Some of our clients don’t want a clinician with a disability so that clinician has a lower caseload but does more administration and supervision. A few other clinicians seem irritated by this.”  “Some people are just angry for no reason on disabled people, so to keep negative attitude away from work environment is definitely a challenging in retaining and promoting disabled people”  “I’m not sure that the managers even have the idea to accept people with disabilities of any kind.”  “Sometimes colleagues can be little harsh on employees with disabilities, they give them extra work thinking they are getting more privileges than them.” |
| Concerns about cost | “The cost of providing accommodation sometimes can go very high depending on the disability of person and it can have a great impact on company’s budget for health aids and it will add extra costs for company.”  “Healthcare coverages sometimes becomes too expensive that become a challenges in promoting disabled people” |
| Concerns about competence | “Employer are afraid that workers with disabilities will not work like other employees.”  “There is fear of not meeting project time quota due to employee with disabilities”  “Some people with disabilities expect employers and coworkers to give them special treatment and support.”  “We’ve has 2 people with disabilities--one was unreliable and the other couldn’t or wouldn’t do the job and was terminated for insubordination such as leaving for home at noon & not returning, pushing work off on others, and reading novels on the job.” |
| Lack of knowledge | “Employers do not know how to handle the needs of a disabled employee.”  “Lack of knowledge of what services and accommodations are required in order to create safe environment for them is quite challenging.”  “Providing accommodation is a headache.”  “Disability awareness training are of no use”  “Lack of information about various disabilities a employee can face.” |
| Concerns about litigation | “Potential lawsuits cause employers to fear they will not be able to discipline those with a disability for poor performance.” |
| *Challenges in retention and promotion | “You’ll find them mistreated at their workplaces they are not easily prepared for promotion”  “Given less authority than other employees.”  “They are not taken into consideration.” |
| *Factors influencing likelihood of overcoming challenges | “People with disabilities could do office work, but unfortunately there are very few such positions.”  “Our company are small scale that’s why we are not providing all full stablished services”  “Depends on the person and the type of disability”  “My industry is looking for a healthy and capable worker.”  “It takes a lot of trouble to hire people with disabilities.”  “Our job requires both professional knowledge and good physical ability. It is not possible to employ people with disabilities.” |
| **Strategies: Employee Resource Groups (ERGs)** | |
| *Positive outcomes | “Our activities foster an environment where qualified individuals with disabilities want to work and where they are respected and valued for the unique talents and skills they bring to our company” |
| *Role of ERGs | “The group acts as ambassador of the company’s commitment to creating a diverse and inclusive workplace”  “We work to create a supportive environment for all employees and to serve as an information source for applicants, recruiters, managers and staff who have questions about disabilities, support and accommodations”  “Making efforts to develop e-learning software to train employees on how to interact with colleagues and customers who are disabled”  “Members use their perspective and first-hand knowledge to educate others about disability etiquette, accessibility and accommodations”  “We created an online resource on the firm’s web site specific to disability awareness to enable our partners and employees to access materials of interest”  “We’ve created facility-accessibility standards that help our facility managers implement signs, lights, cafeterias, reception areas, parking lots, desks and more to help employees and visitors do their best work” |
| Importance of leadership involvement | “Executive champion ensures that our group has visibility among key organizational leaders and influencers and help to secure funding and other resources”  “We provide feedback to executive leadership to make sure our members have equal treatment and opportunities in our organization” |
| **Additional useful strategies** | |
| *Assessing and meeting needs | “Adapting services to meet the diverse needs of persons with disabilities allows business to develop greater flexibility, builds reputations and reaches out to a sizeable market.”  “Understand the need for disabled employees to complete the assigned task.” |
| *Transportation support | “Makes access to public transport easier for employees with multiple disabilities.”  “Public transport should be easily accessible for disabled people.” |
| *Skills matching with job responsibilities | “We promote based upon merit and ability. Those with physical disability are offered accommodation where desired/feasible. Otherwise they are offered training if needed and placement in our nonphysical labor force. All have equal opportunity for and have been promoted.”  “The possibility of employment depends on the type of job for which the candidate applies.”  “Our employees with physical limitations work in the office or in the IT sector, so we have no problem with physical work.” |
| *Establish a supportive work environment | “Creating the right infrastructure for the people with disabilities can be really helpful to retain people.”  “Create a work environment where employees with disabilities feel safe to speak there issues.” |
| *Show respect and appreciation | “Assures disabled employees that they have do have equal value for the employers”  “We make sure to work with anyone and how to approach them with grace and manners with respect. |
| **Importance of workforce diversity and inclusion** | |
| *Positivity about disability inclusion | “As far as I know, we have two employees in the IT sector and we are very satisfied with their abilities, which gives us hope that we will continue to employ people who have a problem.”  “We should put people with disabilities as leaders as I have observed they are quite good in their work and do work with enthusiasm.”  “Diverse work groups develop better solutions to business challenges.”  “Just like others, the majority of persons with disabilities want a dignified and productive life.”  “Many companies have found that by employing persons with disabilities they have been better able to understand and serve their customers with disabilities.”  “It is a great initiative of hiring people with disabilities as it encourages them and they feel positive when they earn for themselves.” |
| *Perspectives not captured by closed-ended questions | |
